# Alzheimer’s disease risk gene *Wwox* controls amyloid pathology through metabolic reprogramming

**DOI:** 10.1093/braincomms/fcag385

**Published:** 2026-10-09

**Authors:** Hannah J Lucas-Clarke, Daniel C Maddison, Leonardo Amadio, Edgar Buhl, Clement Regnault, Owen M Peters, James J L Hodge, Gaynor A Smith

**Affiliations:** Division of Psychological Medicine and Clinical Neurosciences, School of Medicine, Cardiff University, Cardiff CF24 4HQ, UK; UK Dementia Research Institute at Cardiff University, School of Medicine, Cardiff CF24 4HQ, UK; Division of Psychological Medicine and Clinical Neurosciences, School of Medicine, Cardiff University, Cardiff CF24 4HQ, UK; UK Dementia Research Institute at Cardiff University, School of Medicine, Cardiff CF24 4HQ, UK; Division of Psychological Medicine and Clinical Neurosciences, School of Medicine, Cardiff University, Cardiff CF24 4HQ, UK; UK Dementia Research Institute at Cardiff University, School of Medicine, Cardiff CF24 4HQ, UK; School of Biosciences, Cardiff University, Cardiff CF10 3AX, UK; School of Physiology, Pharmacology and Neuroscience, University of Bristol, Bristol BS8 1QU, UK; Shared Research Facilities, College of Medical Veterinary Life Sciences, University of Glasgow, Glasgow G12 8QQ, UK; UK Dementia Research Institute at Cardiff University, School of Medicine, Cardiff CF24 4HQ, UK; School of Biosciences, Cardiff University, Cardiff CF10 3AX, UK; School of Physiology, Pharmacology and Neuroscience, University of Bristol, Bristol BS8 1QU, UK; Division of Psychological Medicine and Clinical Neurosciences, School of Medicine, Cardiff University, Cardiff CF24 4HQ, UK; UK Dementia Research Institute at Cardiff University, School of Medicine, Cardiff CF24 4HQ, UK

**Keywords:** Alzheimer’s disease, WWOX, risk genes, amyloid, *Drosophila*

## Abstract

Genome-wide association studies have identified multiple loci that contribute to late-onset Alzheimer’s disease risk, yet the functional mechanisms of many candidate genes remain unclear. WW-domain containing oxidoreductase (*WWOX*) is one such gene associated with disease and downregulated in patient brains, but its role in neurodegeneration is poorly understood. Here, we use *Drosophila* models to investigate the cell-type-specific and mechanistic roles of *Wwox* in ageing and amyloid-β_42_ (Aβ_42_)-induced pathology. Neuronal and glial knockdown of *Wwox* reduced lifespan and increased sleep drive, indicating a role in brain homeostasis. In an Aβ_42_ transgenic model, neuronal *Wwox* knockdown exacerbated toxicity, shortening lifespan, impairing locomotion, and increasing soluble Aβ_42_ levels. Transcriptomic and metabolomic analyses revealed that *Wwox* knockdown in an Aβ_42_ transgenic model promotes metabolic reprogramming, characterized by elevated lactate dehydrogenase (*Ldh*) expression and increased lactate levels, driven by activation of the stress-responsive transcription factor Atf4. Genetic reduction of *Ldh* rescued locomotor deficits, identifying dysregulated pyruvate metabolism as a key mediator of Wwox-dependent neurotoxicity. Conversely, overexpression of *Wwox* in neurons markedly reduced amyloid burden, improved survival and locomotor function, and reversed Aβ_42_-induced transcriptional changes. Both Aβ_42_ and the protective effects of Wwox were associated with altered amino acid metabolism, including changes in the cysteine and L-methionine pathway. Together, these findings identify WWOX as a regulator of neuronal metabolic state, linking genetic risk to amyloid pathology via metabolic reprogramming, and highlight WWOX activation as a potential therapeutic strategy in Alzheimer’s disease.

## Graphical Abstract

**Graphical Abstract:**
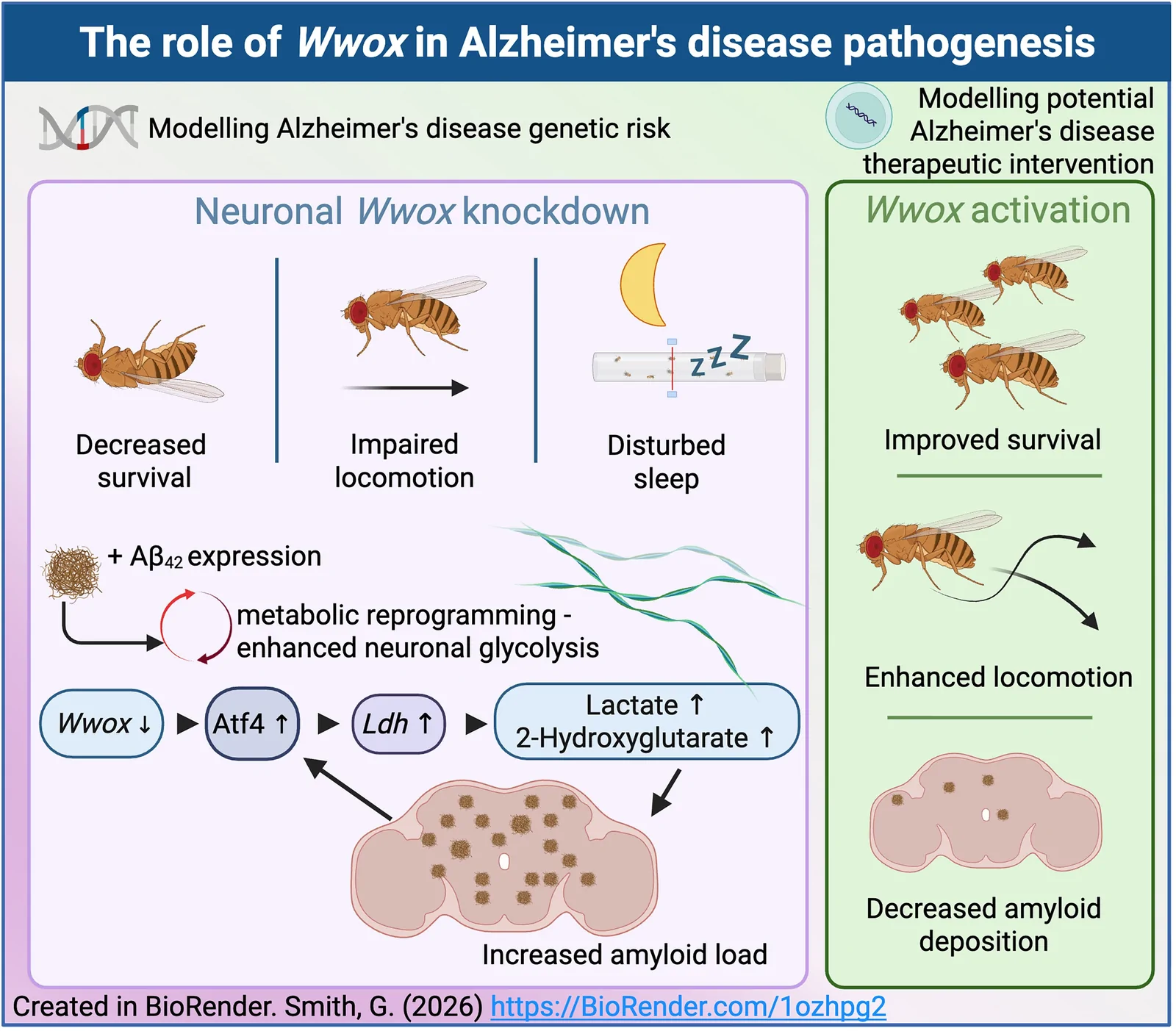

## Teaser

Lucas-Clarke and Maddison *et al*. reported that reduced WW domain-containing oxidoreductase expression increases amyloid toxicity and disrupts metabolism in a fruit fly model of Alzheimer’s disease. Conversely, increasing expression improves survival and reduces amyloid, identifying metabolic changes as a key link between genetic risk and disease progression.

## Introduction

Alzheimer’s disease (Ad) is the most common form of dementia, characterized by pronounced neurodegeneration, causing memory impairment, cognitive decline, and sleep disruption. On a cellular and molecular level, these symptoms follow the accumulation of extracellular amyloid β (Aβ) plaques, hyperphosphorylated tau pathology and reactivity of astrocytes and microglia. Ad manifests in two forms, early- (EOAD) and late-onset (LOAD). Whilst EOAD can be caused by monogenic mutations in amyloid precursor protein (*APP*), presenilin-1 (*PSEN1*) or presenilin-2 (*PSEN2*), LOAD is considered sporadic and a result of the convergence of environmental and genetic risk factors. Single nucleotide polymorphisms (SNPs) at ∼90 genomic loci have been discovered to contribute to LOAD risk.^1^ Determining how genes associated with these SNPs contribute to disease onset and progression is vital to our understanding of LOAD and how it may be prevented or treated.

An intergenic SNP located on chromosome 16 between WW domain-containing oxidoreductase (*WWOX*) and MAF bZIP transcription factor (*MAF*) has been associated with an increased the risk of developing LOAD.^2^ Genome-wide expression quantitative trait locus (eQTL) mapping between LOAD patients and controls identified *WWOX* as a significant eQTL in the brain with a negative *β* value, indicating a decrease in *WWOX* mRNA in individuals with the associated SNP.^3^ A genome-wide significant association has also been made between a SNP within a predicted *WWOX* enhancer region and a key disease biomarker, Aβ_42_/Aβ_40_ ratio and decreased WWOX has been observed in post-mortem hippocampal tissue of Ad patients compared with controls.^4,5^ Interestingly, WWOX has been implicated in a protein aggregation cascade and may influence the hyperphosphorylation of tau.^6-8^ However, the mechanisms underlying *WWOX* modulation of Ad pathology are not well understood.

*WWOX* is an oxidoreductase and tumour suppressor that plays a significant role in cancer cell metabolism, influencing glycolysis, mitochondrial integrity, signalling and oxidative stress.^9-13^ It contains two N-terminal WW domains, characterized by highly conserved adjacent tryptophan residues, binding to proline-rich regions in protein ligands and is thus responsible for the mediation of interactions between WWOX and multiple protein partners.^14^ In addition, *WWOX* loss of function has been postulated to mediate a Warburg-like effect, a switch to glycolysis despite aerobic conditions, via modulation of HIF1α, a transcription factor with a variety of metabolic targets including glucose transporter 1 (*GLUT1*) and lactate dehydrogenase (*LDH*).^9,15,16^ A similar metabolic shift has been observed in *Drosophila* and human stem cell-derived neuronal models of Ad,^17,18^ but whether this is influenced by WWOX, in the context of Ad, has not yet been explored. Thus far WWOX has only been linked to glycolysis in fibroblasts, cancer cells and peripheral metabolic tissues.^9-13^

We sought to address this question by altering *Wwox* levels in the *Drosophila melanogaster* brain, via neuronal or glial-specific RNA interference (RNAi) and CRISPR activation (CRISPRa). We explored the age associated effects on Ad-relevant phenotypes with or without the overexpression of the human Aβ_42_ peptide.^19^ We show that both neuronal and glial knockdown of *Wwox* shortened lifespan and increased sleep, in the absence of Aβ_42_. However, in the presence of Aβ_42,_  *Wwox* knockdown exacerbates lifespan and locomotor deficiencies in a neuron-specific manner, whilst also increasing amyloid load. Transcriptomic and metabolomic analyses identified that elevated *Ldh*, as a consequence of Aβ_42_ overexpression, was exacerbated by *Wwox* knockdown and was accompanied by increased levels of lactate and the integrated stress response (ISR)/unfolded protein response (UPR) regulator Atf4. Strikingly, upregulation of *Wwox* in neurons significantly reduced amyloid load, rescuing lifespan and locomotor defects but did not alter lactate levels, instead altering L-methionine and reversing the transcriptional dysregulation seen in the Aβ_42_ model. Taken together, these findings provide insight into the mechanisms underlying LOAD risk conferred by genetic variation at the *WWOX* locus and identify WWOX activation as a strategy for reducing Aβ_42_ pathology.

## Materials and methods

### Fly husbandry

*Drosophila* stocks (Supplementary Table 1A) were housed at room temperature and transferred to fresh standard ‘Cornmeal-Molasses-Yeast’ media, set with agar, once every 3 weeks. Crosses and experimental lines (reported in Supplementary Table 1B for each experiment) were housed at 25°C on a 12:12 h light-dark schedule.

### Evaluation of *Wwox* expression in a single cell atlas of the ageing *Drosophila* brain

*Wwox* expression was assessed using a single cell transcriptomic dataset of the ageing *Drosophila* brain available on SCOPE.^20^ We calculated the percentage of cells within annotated clusters that expressed detectable levels of *Wwox*.

### Lifespan

Newly eclosed females (Supplementary Table 1B) were allowed to mate for <48 h, then were separated into vials of *n* < 11 and passed to fresh food two to three times a week, whilst scoring deaths. Lifespan and other phenotypic analyses were not directly compared with males because of the hyper X activation effects.^21^ Alive flies that did not successfully transfer into a new vial were included as censored flies in the analysis. Kaplan-Meier plots were generated using GraphPad Prism v.10.

### Rapid iterative negative geotaxis (RING)

Newly eclosed females (Supplementary Table 1B) were allowed to mate for <48 h and then divided into vials of *n* < 11. Progeny were flipped onto fresh food two to three times a week and assayed at 7-, 14-, 21- and 28-days post eclosion (±1 day). After 5 min of acclimatization, the apparatus was raised to a fixed height (8 cm) and dropped, images were captured once a second for 10 s. After 1 min recovery, this procedure was repeated for a total of five drops. Using FIJI, the mean distance climbed by each fly at the 4 s time point was measured.

### Assessment of sleep and circadian rhythms

Males (0–6 days, Supplementary Table 1B) were individually housed within *Drosophila* activity monitors (DAM, Trikinetics Inc) and subjected to a 12-h light:dark (LD) cycle at 25°C for 5 days, followed by 5 days of 24-h darkness (DD). For each run (>2 runs), *N* = 31–80 individual males were assayed per genotype. Activity (beam crosses) was calculated in 1- and 30-minute bins using DAMFileScan v1.11 and an in-house script (https://github.com/Fly-Cardiff/sleep_analysis) and subsequently analysed using ‘Sleep and Circadian Analysis MATLAB Program—SCAMP’ (MATLAB, vR2020b).^22,23^ A bout of sleep was defined, as per convention, as more than 5 min without activity.^24,25^ Data from the LD phase was derived after exclusion of day one, to allow for entrainment, and after removal of any deaths.

Seventeen metrics of sleep and wakefulness were exported from SCAMP for subsequent analyses: total wake episodes, mean wake episode duration, total activity counts, total time awake, latency to sleep, mean wake duration minus latency, mean activity a minute whilst moving, activity normalized to time awake, the probability of transitioning to an inactive state or active state, the number of sleep episodes, the total, max, mean and median sleep episode duration and sleep stability. All metrics were assessed for inter-correlations (Pearson) and strongly correlated (*r* > |0.8|) metrics were excluded from further analysis. Of the remaining metrics, normality of residuals was assessed with Kolmogorov-Smirnov tests and quantile-quantile plots and consequent Box-Cox transformations were performed where data was deemed sufficiently non-gaussian (RStudio v2022.12, GraphPad Prism v.10).

Assessment of circadian strength was performed for the DD regime, including flies that survive to day ten. Rhythmicity statistic (RS) and period length were derived from SCAMP autocorrelograms. The RS describes the ability of a fly to maintain a cycle, with an RS > 1.5 being rhythmic, RS = 1–1.5 being weakly rhythmic and flies with an RS < 1 being arrhythmic. Periodicity was calculated for rhythmic and weakly rhythmic flies only. If a fly had a RS < 0, it was set to zero for analysis of rhythmicity.

### Immunohistochemistry

Day 14 *Drosophila* heads were dissected and fixed in 4% PFA in PBS with 0.1% Triton X-100 (PTX) for 10 min (Supplementary Table 1B). Brains were then dissected from the head capsule and fixed for a further 10 min. Brains were blocked in 10% normal goat serum (in PTX) for 1 h at room temperature, then incubated with primary antibodies—anti-Aβ_42_ (1–16, 6E10, mouse, 1:400, BioLegend #803001), anti-Atf4 (rat, 1:200, gifted by Bateman lab)^26^—for 60 and 16 h respectively at 4°C in 10% normal goat serum (in PTX). Brains were washed five times in PTX, then incubated with fluorophore-conjugated secondary antibodies AlexaFluor-647 (anti-mouse, 1:500, Invitrogen #A-21235), AlexaFluor-594 (anti-rat, 1:400) for 1–2 h at room temperature in PTX. Brains were then washed five times in PTX, and then mounted in VECTASHEILD Antifade (Vector Laboratories, H-1000-10) before imaging on a Cell Observer Spinning Disk Confocal Microscope (ZEISS) with Plan Apo 20X/0.8 dry objective and Axiocam 503 monochromatic camera, using Zen Blue software (ZEISS). Images were processed in FIJI—Aβ aggregates were detected by the *Analyze Particles* function and Atf4 fluorescence intensity (mean grey value) was averaged across four equal sized region of interests (ROIs, 227.5 µm^2^) per brain. Data was analyzed with Mann-Whitney tests or *t*-tests dependent on normality (GraphPad Prism v.10).

### Imaging of sima:GFP fluorescent reporter

Brain sima levels were measured using the genetically encoded reporter line *UAS-sima:GFP* in day 14 flies (Supplementary Table 1B). Unfixed brains were dissected and imaged on a Zeiss Cell Observer spinning disk confocal using 488 nm excitation, Plan Apo 20X/0.8 dry objective and Axiocam 503 camera. Fluorescence intensity (mean grey value) was averaged across four equal sized (415.1 µm^2^) ROIs (FIJI).

### Electrochemiluminescence quantification of Aβ_42_

Day 14 flies were snap-frozen in liquid nitrogen and heads (*n* = 25 per replicate) separated by vortexing (Supplementary Table 1B). Heads were then homogenized for 3 min in 150 µL of soluble extraction buffer (50 mM HEPES pH 7.3, 5 µM EDTA and 1X protease inhibitor) on ice, vortexed, then incubated for 10 min at room temperature. Homogenates were sonicated at 4°C for 4 min (30 s on 30 s off), then centrifuged at 21 000 × g for 5 min at 4°C. The supernatant was separated and used as the soluble fraction. 55 µL of insoluble extraction buffer (soluble extraction buffer with 5 mM Guanidinium HCl) was added to the remaining pellet. The pellet was then homogenized for 2 min, vortexed, and incubated for 10 min at room temperature. Homogenates were sonicated in a 4°C water bath for 4 min (30 s on 30 s off), then centrifuged at 21 000 × g for 5 min at 4°C. The supernatant was separated and used as the insoluble fraction.

The Meso Scale Discovery (MSD) V-plex assay was performed according to manufacturer’s instructions. Briefly, the plate was blocked with diluent 35 for 1 h at room temperature. Standard calibrators were serial diluted in Diluent 35. After blocking, the plate was washed three times with 150 μL/well of 0.05% Tween-PBS. Next, 25 μL of detection antibody (anti-Aβ 17–24, 4G8, mouse, MSD) was added to each well, followed by 25 µL of standard or 0.5 μg/µL brain homogenate from soluble or insoluble fractions. The plate was sealed and incubated whilst shaking for 2 h. The plate was subsequently washed six times with 150 μL/well of 0.05% Tween-PBS. The final wash was discarded and 150 μL of 2× read buffer was added to each well, before immediately reading the plate on an MSD instrument.

### Bulk transcriptomics

Heads of 14-day old, mated females (*n* = 10, Supplementary Table 1B) were removed and pooled into *N* = 4 replicates per genotype. RNA extracted using the RNAqueous-Micro kit (AM1931) according to the manufacturer’s instructions and sent to the Cardiff University School of Biosciences Genomics Research Hub for 6.5 million reads paired-end mRNA sequencing on an Illumina NextSeq500 with a fragment length of 75 base-pairs. Quality was screened using FastQC (0.11.8); Poly-G tails, Truseq3 adapters and low-quality bases/regions (phred <3 or sliding window average of <15) were trimmed using Illumina clip (Trimmomatic).^27^ Any remaining reads <20 bases in length were also removed. Reads were mapped to the *Drosophila melanogaster* genome (BDGP6.32) using STAR and reads counted using featureCounts.^28,29^ Non-coding RNAs, transposons and unassigned features were removed using SARTools.^30^ Differential gene expression analysis was then performed using DESeq2 with log_2_ fold-change shrinkage using the ashr algorithm.^31,32^ Dimensionality of variance stabilized data was assessed via principal component analyses (PCA, prcpomp). Volcano plots were generated plotting −log_10_ adjusted *P*-value against log_2_ fold-change (threshold = ±0.58) (ggplot2, RStudio v.4.4.1).^33^ Over representation analysis was performed between differentially expressed genes and gene ontology molecular function (GO:MF), gene ontology biological process (GO:BP), KEGG, Wiki Pathways and TRANSFAC databases, via the functional enrichment tool g:Profiler (version e112_eg59_p19_25aa4782).^34,35^

### Metabolomics

Heads of 14-day old, mated females were removed and separated into *N* = 5 replicates of *n* = 10 (Supplementary Table 1B). Metabolites were extracted by homogenizing in 1:3:1 chloroform (SLS, C2432), methanol (Fisher, 10284580) and water, followed by an hour agitation at 4^°^C. Hydrophilic Interaction Liquid Chromatography (HILC) based metabolomics was performed (UltiMate 3000 RSLC, Thermo Fisher Scientific) using a hydrophilic ZIC-pHILIC column (Merck) on samples and standards (Metabolomics Standards Initiative). Samples, were ionized using a Thermo Orbitrap QExactive (Thermo Fisher Scientific) and mass spectrometry performed in both ionization modes, detecting metabolites in range of 70–1050 m/z. Data processing, feature annotation (based on exact mass match to compounds in databases) and identification (based on exact mass and retention time match to an authentic standard) was performed using IDEOM, mzMatchISO, PiMP and Metaboanalyst 5.0.^36-38^ Fragmentation analysis of the pooled samples was performed using in-house software FrAnK.

### Statistical analysis

Normality was assessed using Kolmogorov-Smirnov tests and quantile-quantile plots. Dependent on normality and the number of experimental groups, different tests were performed with an *α* = 0.05. For data deemed Gaussian with just two groups, unpaired two-tailed *t*-tests were performed. If data was matched and two experimental variables were being assessed, we performed mixed effect models. If two experimental variables were being assessed, but unmatched, we performed two-way ANOVAs. If just one experimental variable was being assessed, with more than two groups, we performed one-way ANOVAs. Significant effects, where appropriate, were explored further using Šidák, Tukey’s, Dunnett’s or Bonferroni multiple comparisons, dependent on the effect being explored. If multiple tests were performed, *P* values were further corrected with 5% false discovery rate (FDR). If data was deemed non-Gaussian we performed non-parametric Mann-Whitney tests. Lifespan was analysed using Mantel-Cox and Cox proportional hazard-model tests (GraphPad Prism v.10).

RNA sequencing was analysed with DESeq2 with Fisher Exact Tests to assess similarities between gene sets using the GeneOverlap package.^32,35^ Pearson correlations were used to assess the relationship between log_2_ fold-changes. Log-transformed relative metabolite intensities were analysed via two-way ANOVAs on the top 200 features (based on variance) followed by multiple testing correction with 10% FDR using Metaboanalyst 5.0.^37^

## Results

### *Wwox* knockdown in neurons or glia shortens lifespan

WWOX is highly conserved between species, with *Drosophila Wwox* sharing 65% sequence similarity with human *WWOX*, and a score of 16/16 using the DRC Integrative Ortholog Prediction Tool (DIOPT).^39^ Equivalent to its mammalian orthologue,^40^  *Drosophila Wwox* is expressed throughout the brain. Using a single cell transcriptomic dataset from the ageing *Drosophila* brain,^20^  *Wwox* expression was detectable in both neuronal and glial cell-types (Fig. 1A).

**Figure 1:**
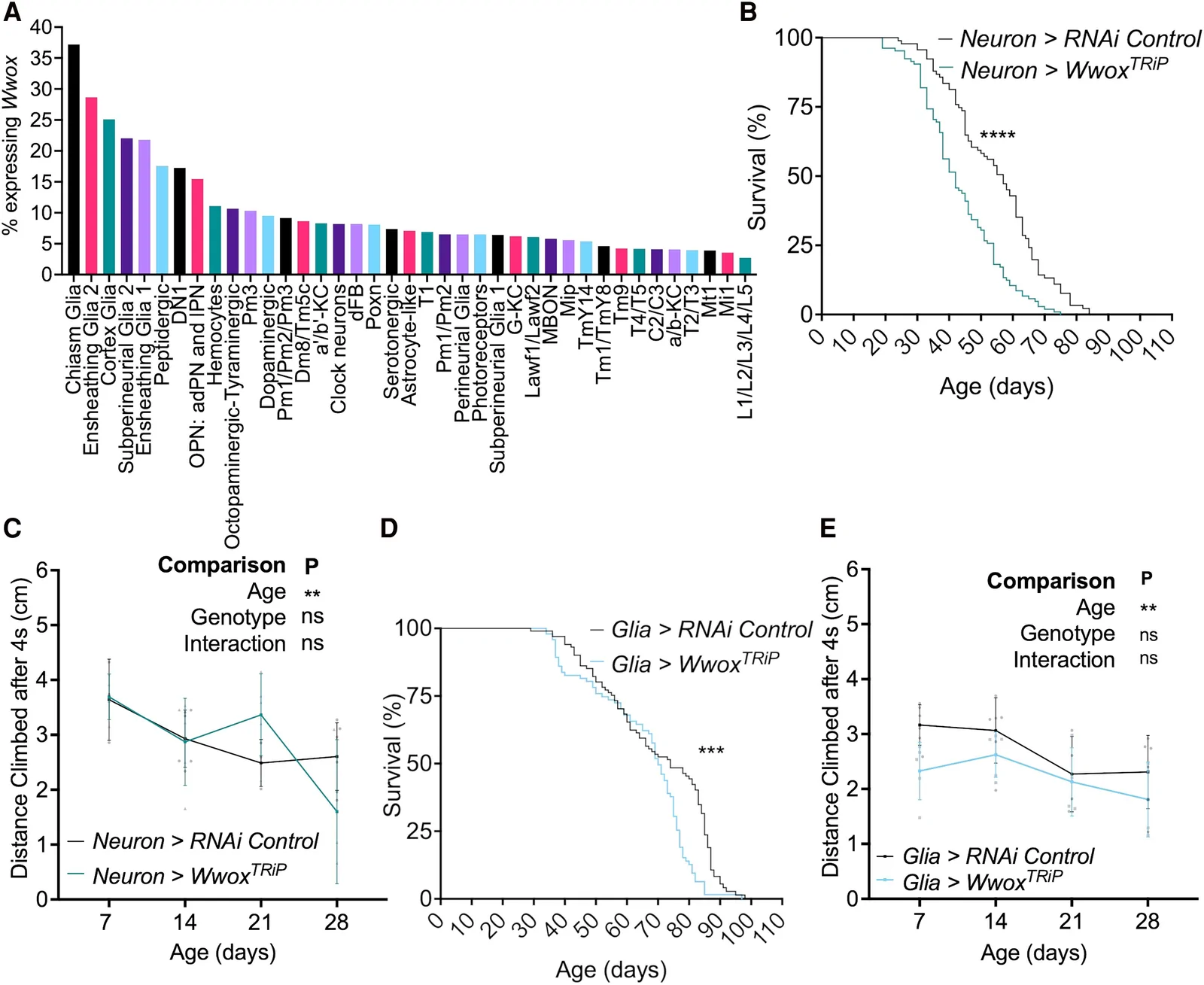
**Knockdown of neuronal and glial *Wwox* reduces longevity.** (**A**) Analysis of single cell RNAseq data acquired through SCOPE,^20^ demonstrates that *Wwox* is expressed in both glia and neurons in the *Drosophila* brain. Data is presented as percentage of cells from each cluster expressing detectable levels of *Wwox* mRNA. Total number of cells in the whole dataset was 57 000 from *N* = 40 adult brains. (**B**) Lifespan is reduced upon pan-neuronal *Wwox* knockdown (*N* = 91–105 per genotype). (**C**) Distance climbed after 4 seconds (s) in a startle induced locomotion assay (7-, 14-, 21-and 28-day old flies) is unaffected by neuronal *Wwox* knockdown at (*N* = 3–6, *n*∼10 flies per N). (**D**) Glial knockdown of *Wwox* reduces median lifespan (*N* = 98–101). (**E**) Startle induced locomotion (7-, 14-, 21-and 28-day old flies) is unaffected by glial knockdown of *Wwox* (*N* = 4–6, *n*∼10 flies per N). Statistics = Lifespan was assessed via Mantel-Cox tests. Locomotion was assessed via mixed effect models (matching by vial, panel E) or two-way ANOVA with factors of age and genotype, followed by Šidák multiple comparisons (panel C). Data is represented as mean ± SD. For lifespan (panels B and D) *N* represents biological replicates of individual flies. For locomotion (panels C and E) *N* represents independent vials (data points plotted) of *n* individual flies (females). *P < 0.05, ** < 0.01, *** < 0.001, **** < 0.0001, ns = non-significant. (panel A): DN1 = Dorsal clock neurons 1, OPN = olfactory projection neurons, adPN = antero-dorsal olfactory projection neurons, lPN = lateral olfactory projection neurons, Pm1/Pm2/Pm3 = proximal medulla neurons 1/2/3, Dm8 = Distal medulla neurons 8, Tm5c = transmedullary 5c neurons, a/b-KC = α/β lobe kenyon cells, a′/b′-KC = α′/β′ lobe kenyon cells, G-KC = γ lobe kenyon cells, dFB = dorsal fan-shaped body neurons, T1 = T1 neurons, Lawf1/2 = lamina wide-field neurons 1/2, MBON = mushroom body output neurons, Mip = Myoinhibitory peptide neurons, TmY14 = transmedullary Y 14 neuron, Tm1 = transmedullary 1 neuron, TmY8 = transmedullary Y 8 neuron, Tm9 = transmedullary 9 neuron, T4/T5 = T4/T5 neurons, C2/C3 = centrifugal interneurons 2/3, Mt1 = medulla tangential 1, Mi1 = Medulla intrinsic neuron, L1/L2/L3/L4/L5 = lamina monopolar neurons 1/2/3/4/5.

To explore the cell-type specific roles of *Wwox* and mimic the subtle expression changes observed in Ad, we expressed a *Wwox* targeted RNAi construct under control of *elav-GAL4* (pan-neuronal) or *repo-GAL4* (pan-glial) drivers. *Wwox* knockdown in neurons (Supplementary Fig. 1),^10,11,41^ significantly reduced lifespan in comparison to control (median lifespans = 57 and 42 days respectively, *N* = 91–105, χ^2^ = 30.76 *P* < 0.0001 Fig. 1B). Startle-induced locomotor function was significantly decreased with age (*F* = 6.106, *P* = 0.0026) but unaffected by neuronal knockdown of *Wwox* (*F* = 0.018, *P* = 0.8942) or the interaction between the two (*F* = 2.316, *P* = 0.0982, Fig. 1C, *N* = 3–6). Glial knockdown of *Wwox* caused a reduction in lifespan in comparison to control (median lifespan = 70 versus 74 days, *N* = 98–101, χ^2^ = 13.07 *P* = 0.0003, Fig. 1D), albeit milder than that of neuronal knockdown. Like neuronal knockdown, age significantly worsened startle-induced locomotor activity (*F* = 7.454, *P* = 0.0033), however glial knockdown of *Wwox* had no effect (*F* = 3.830, *P* = 0.0820), nor was there an interaction (*F* = 0.7327, *P* = 0.5142, Fig. 1E, *N* = 4–6). These results suggest that both neuronal and glial *Wwox* play a role in longevity but are dispensable for startle-induced locomotor function up to 28 days old.

### *Wwox* knockdown in neurons or glia decreases wakefulness and increases sleep

A common symptom of Ad is disruption to circadian rhythms and sleep homeostasis,^42-46^ leading us to next to assess the contribution of neuronal or glial *Wwox* expression to sleep and circadian activity regulation. Sleep metrics were derived from activity of individually housed flies using the MATLAB toolbox SCAMP.^22,23^ The effect of *Wwox* manipulation was investigated on: total activity counts, activity counts normalized to time awake, max wake episode duration, mean wake episode duration, sleep latency, probability to transition from an active to inactive state (sleep pressure) and number of sleep episodes. Other strongly correlated sleep metrics (*r* > |0.8|) are shown in Supplementary Table 2.

Neuronal knockdown of *Wwox* decreased activity normalized to time awake in the light phase (*t* = 4.969, *P*_adj_ < 0.0001, Fig. 2A) and increased sleep pressure (*t* = 7.146, *P*_adj_ < 0.0001, Fig. 2B), whilst increasing mean sleep episode duration, both in the light and dark phase (*t* = 8.978 and *t* = 6.066 respectively, both *P*_adj_ < 0.0001, Fig. 2C, *N* = 31–80 over >2 runs). Similarly, pan-glial *Wwox* knockdown reduced activity normalized to time awake (*F* = 29.81, *P*_adj_ < 0.0001, Fig. 2D), increased sleep pressure in the light and dark phases (*t* = 7.268 *P*_adj_ < 0.0001, *t* = 2.870 *P*_adj_ = 0.0129, respectively, Fig. 2E) and increased mean sleep episode duration (*F* = 5.768, *P*_adj_ = 0.0258, Fig. 2F, *N* = 53–56 over >2 runs). Additional sleep metrics are summarized in Supplementary Fig. 2A–J. To understand whether these changes in sleep were due to underlying circadian differences, we evaluated the periodicity of flies in constant darkness without light entrainment cues. After neuronal knockdown of *Wwox*, all flies remained strongly rhythmic (*t* = 0.1766, *P* = 0.8606, Fig. 2G, *N* = 17–34) with circadian periods comparable to control (*U* = 178, *P* = 0.8329, Fig. 2H and I, *N* = 12–31). Glial knockdown of *Wwox* had no effect on rhythmicity (*t* = 1.058, *P* = 0.2955, Fig. 2J, *N* = 6–42) or period length (*U* = 50, *P* = 0.3052, Fig. 2K and L, *N* = 4–37). *Wwox* knockdown driven by the clock specific driver *timeless-GAL4*, resulted in a similar decrease in wakefulness (Supplementary Fig. 2K–R) to pan-neuronal and pan-glial knockdown, with flies remaining strongly rhythmic (Supplementary Fig. 2S) with a slightly increased circadian period (Supplementary Fig. 2T). These results suggest that *Wwox* knockdown, irrespective of cell type, increases sleep, without disrupting circadian rhythmicity.

**Figure 2:**
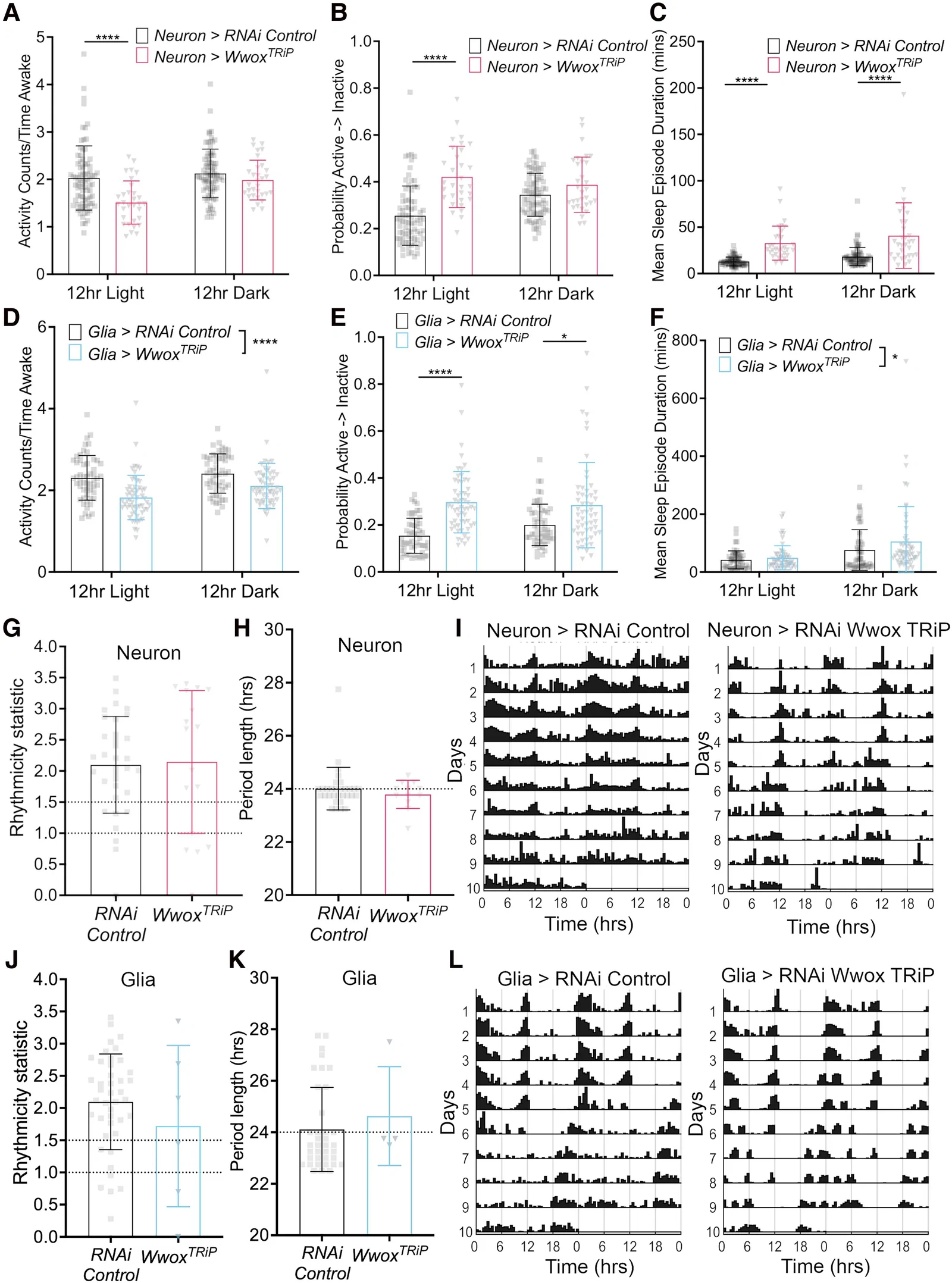
**Knockdown of *Wwox* decreases wakefulness and increases sleep.** Pan-neuronal *Wwox* knockdown decreases (**A**) locomotor activity, (**B**) increases sleep pressure and (**C**) increases mean sleep episode duration in minutes (mins). (**D**) Pan-glial *Wwox* knockdown decreases locomotor activity, (**E**) increases sleep pressure and (**F**) increases mean sleep episode duration. Sleep metrics were collected over *n* > two independent runs, *N* = 31–80 individual flies per genotype. (**G**) Rhythmicity statistic (RS) and (**H**) period length of circadian behaviour was unaffected upon pan-neuronal *Wwox* knockdown in dark conditions. (**I**) Representative double plotted actograms over 24 hours (hrs) for pan-neuronal *Wwox* knockdown. (**J**) RS and (**K**) period length were unaffected upon pan-glial *Wwox* knockdown. (**L**) Representative actograms for pan-glial *Wwox* knockdown. Dotted lines at RS = 1 (weakly rhythmic threshold), RS = 1.5 (strongly rhythmic threshold) and period length = 24 h. Circadian rhythmicity was analysed from *N* = 17–34 (panel G) and *N* = 6–42 (panel J) individual flies per genotype, due to removal of deaths. Whereas circadian period was assessed from *N* = 12–31 (panel H) and *N* = 4–37 (panel K), due to the further removal of arrhythmic flies. Statistics = Sleep metrics were analysed via two-way ANOVA followed by Bonferroni multiple comparisons, further corrected for multiple comparisons across all sleep metrics by 5% FDR. *P*_adj_ * < 0.05, **** < 0.0001. Circadian data was analysed via two-tailed unpaired *t*-test or non-parametric Mann-Whitney tests (non-significant). Data points represent individual flies (0–6 day old males), mean ± SD.).

As Wwox is an oxidoreductase and has been linked to alterations in reactive oxygen species (ROS) formation,^10,11^ which in turn can alter sleep homeostasis,^47,48^ we explored whether the sleep alterations witnessed in our model were due to increases in ROS. utilizing the roGFP2-Orp1 reporter for mitochondrial hydrogen peroxide, we witnessed an increase in ROS after neuronal *Wwox* knockdown (*t* = 3.221, *P* = 0.0047, Supplementary Fig. 2U, *N* = 9–11)—suggesting that *Wwox* knockdown may increase sleep, at least in part, through an increase in ROS.

### *Wwox* knockdown modifies Aβ_42_-induced phenotypes and increases Aβ_42_ load

Next, we explored the impact of *Wwox* knockdown on amyloid pathology, a hallmark feature of Ad. We employed a model of amyloid toxicity whereby neuronal overexpression of aggregate-prone human Aβ_42_ harbouring the Arctic mutation (E693G) and secretion signal-peptide from the argos gene, drives secretion of the peptide to the extracellular milieu. This causes accumulation of amyloid deposits, leading to brain vacuolation, locomotor deficits and reduction in lifespan in the fly.^19^ Neuronal knockdown of *Wwox* combined with overexpression of human Aβ_42_ did not affect expression of Aβ_42_ mRNA (Supplementary Fig. 3A), but caused a further reduction in lifespan compared with Aβ_42_ expression alone (median lifespan = 41 versus 26 days, χ^2^ = 103.7, *P*_adj_ < 0.0001, Fig. 3A, *N* = 90–99). Cox-proportional hazard modelling of the effects revealed a significant interaction between Aβ_42_ overexpression and *Wwox* knockdown (*Z* = 4.815, *P* < 0.0001), indicating that the decrease in lifespan is not simply the additive effect of the two variables but synergistic and that *Wwox* knockdown is modulating amyloid toxicity directly. As expected, startle-induced locomotor ability was significantly reduced with age (*F* = 11.77, *P* < 0.0001, Fig. 3B), from ∼3.5 cm climbed at day 7 compared with only ∼1.0 cm climbed at day 28 in RNAi Control Aβ_42_ flies. *Wwox* knockdown led to a significant reduction in locomotion (*F* = 12.51, *P* = 0.0019, Fig. 3B), climbing just ∼0.5 cm by day 21 (representative images available in Supplementary Fig. 3B). We found no significant interaction between age and genotype (*F* = 0.3165, *P* = 0.8133, Fig. 3B, *N* = 3–5 vials), suggesting that *Wwox* knockdown in the presence of amyloid significantly worsened locomotor ability at all four time points assessed. Utilising a dual-binary transgene expression system, we restricted *Wwox* knockdown to glial cells (*repo-GAL4*), whilst simultaneously restricting Aβ_42_ expression to neurons (*nSyb-QF2*). *Wwox* knockdown mildly, albeit significantly, decreased lifespan (median = 22 versus 24 days, χ^2^ = 6.720 *P* = 0.0095, Supplementary Fig. 3C, *N* = 100–115) but had no effect on startle-induced locomotion (*t* = 0.9876, *P* = 0.3491, Supplementary Fig. 3D, *N* = 5–6 vials). These results suggest that neuronal *Wwox* may be more important than glial *Wwox* for modulation of Aβ_42_ toxicity.

**Figure 3:**
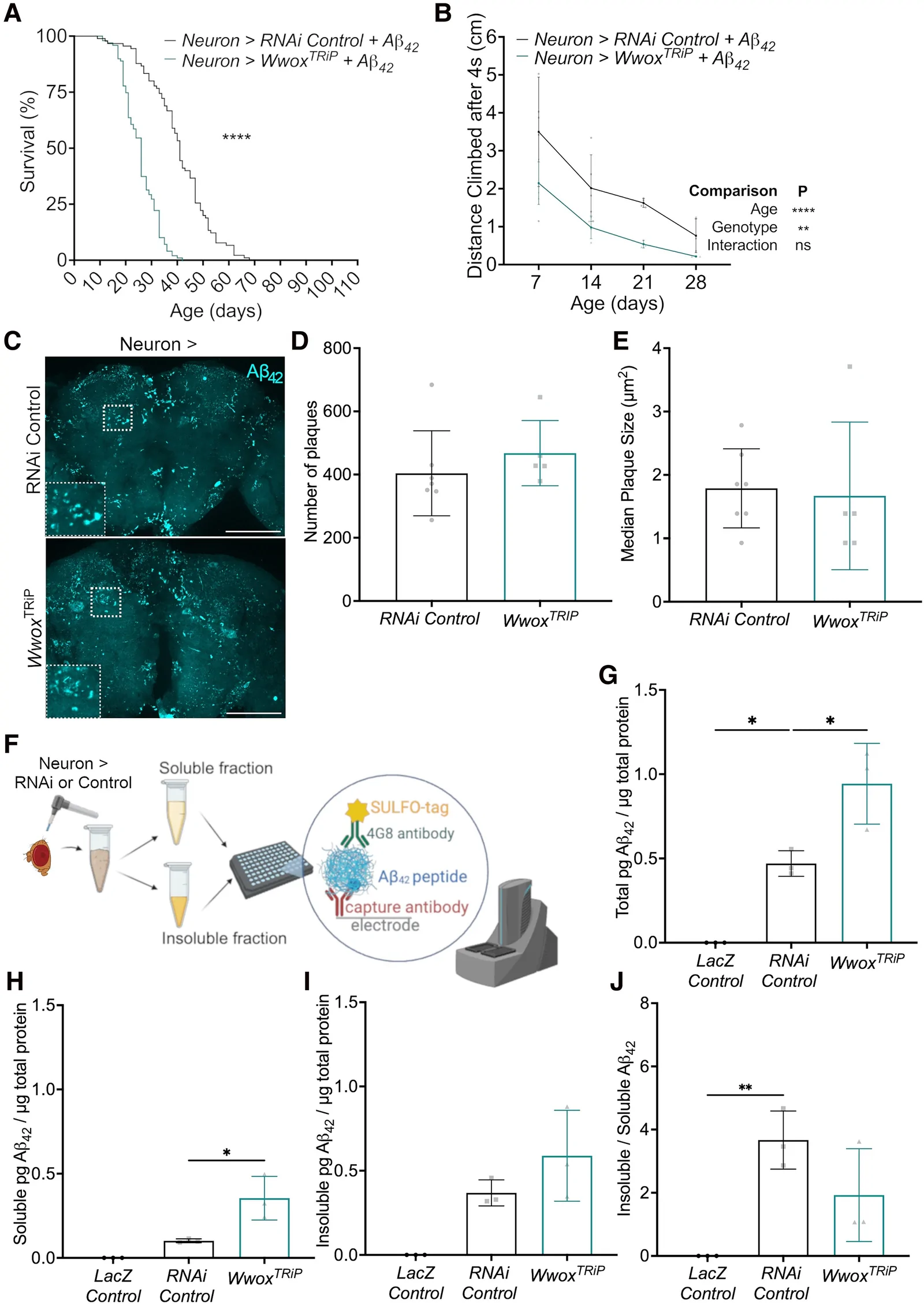
***Wwox* knockdown exacerbates Aβ_42_ related phenotypes.** (**A**) Lifespan is reduced upon pan-neuronal *Wwox* knockdown in the presence of Aβ_42_ overexpression (*N* = 45–68 individual flies). (**B**) Distance climbed after 4 seconds (s) in a startle induced locomotion assay (7-, 14-, 21-and 28-day old flies) is reduced upon pan-neuronal *Wwox* knockdown in the presence of Aβ_42_ overexpression (*N* = 3–5 vials of *n*∼10 flies). (**C**) Aβ_42_ immunocytochemistry (cyan) from day 14 *Drosophila* brains upon *Wwox* knockdown in neurons. (**D**) Quantification of Aβ_42_ staining revealed no changes in number (*N* = 5–7 brains) or (**E**) plaque size (*N* = 5–7 brains) after neuronal *Wwox* knockdown. (**F**) Schematic of Aβ_42_ quantification in head soluble and insoluble fractions by MSD assay (day 14). Created in BioRender. Maddison, D. (2026) https://BioRender.com/d4bip72. (**G**) Total and (**H**) soluble Aβ_42_ levels quantified by MSD assay increase with *Wwox* knockdown in neurons (*N* = 3 pooled replicates of *n* = 25 heads each). **(I**) But identified no changes in insoluble or (**J**) the insoluble/soluble ratio (*N* = 3 pooled replicates of *n* = 25 heads each). Statistics = Lifespan was analysed by Mantel-Cox and Cox proportional hazard tests. Startle-induced locomotion was analysed by two-way ANOVA with factors of age and genotype (Šídák's multiple comparisons correction). Plaque number was assessed by Mann-Whitney tests and size by unpaired *t*-test. MSD assay was analysed by one-way ANOVA followed by Dunnett’s multiple comparisons tests *P*/*P*_adj_ * < 0.05, ** < 0.01, *** < 0.001, **** < 0.0001. Mean ± SD. Scale bar = 100 µm. All experiments were performed on females. Datapoints represent *N*: independent vials (panel B), independent brains (panels C–E), independent pools of brains (panels G–J).

To understand if neuronal *Wwox* knockdown directly impacts endogenous amyloid accumulation, we probed intact brains from day 14 flies using the anti-Aβ [6E10] antibody, choosing this time-point due to clear locomotor defects but before a substantial proportion of flies were dead. Specificity of the immunostaining was confirmed by lack of visible deposits in LacZ-expressing control brains at the same timepoint (Supplementary Fig. 3E). Visible Aβ_42_ deposits were quantified in Aβ_42_-expressing flies, however no significant changes in aggregate number (*U* = 9, *P* = 0.2020, Fig. 3C and D) or median size (*t* = 0.2313, *P* = 0.8218, Fig. 3C and E, *N* = 5–7) were observed upon *Wwox* knockdown. To quantify changes in Aβ_42_ deposition with increased sensitivity, we assessed soluble and insoluble fractioned extracts of 14-day old fly heads by an electrochemiluminescence immunoassay (MSD) using the anti-Aβ [4G8] antibody (Fig. 3F). Total Aβ_42_ was significantly increased upon neuronal *Wwox* knockdown (*q* = 3.997, *P*_adj_ = 0.0127 Fig. 3G). Interestingly, this effect was led by an increase in soluble Aβ_42_ (*q* = 4.126, *P*_adj_ = 0.011 Fig. 3H), whereas insoluble fractions were non-significantly changed (*q* = 1.666, *P*_adj_ = 0.2412, Fig. 3I). However, the ratio between soluble/insoluble Aβ_42_ was not significantly changed (*q* = 2.131, *P*_adj_ = 0.1307, Fig. 3J, *N* = 3). Taken together, these results indicate an increase in Aβ_42_ load upon neuronal *Wwox* knockdown, without an associated increase in detectable aggregate size or number.

### Neuronal *Wwox* knockdown in the presence of Aβ_42_ alters pyruvate metabolism

To investigate the mechanisms underlying the effect of neuronal *Wwox* knockdown on Aβ_42_ associated phenotypes, we performed bulk transcriptomics from flies overexpressing Aβ_42_ or a LacZ control construct, with or without neuronal *Wwox* knockdown (*N* = 4 replicates of *n* = 10 pooled heads). Transcriptomics were performed at day 14, an age at which the flies showed a clear motor phenotype but before significant mortality. The effects of *Wwox* knockdown and Aβ_42_ expression could be clearly separated along PC1 and PC2, explaining 23% and 15% of variance respectively (Supplementary Fig. 4). Knockdown of *Wwox* in a control background significantly downregulated 54 genes and upregulated 44 (Fig. 4A, Supplementary Table 3). Overrepresentation analysis determined these genes were enriched for oxidoreductase activity and metabolism of ascorbate, aldarate, and porphyrins (Fig. 4B, Supplementary Table 3). Overexpressing human Aβ_42_ in neurons downregulated 23 genes and upregulated 51 (Fig. 4C, Supplementary Table 3); overrepresentation analysis revealed a significant association of these genes with fatty acid and pyruvate metabolism (Fig. 4D, Supplementary Table 3), suggesting amyloid expression induces metabolic changes. Knockdown of *Wwox* in neurons also overexpressing Aβ_42_, significantly downregulated 58 genes and upregulated 54 genes (Fig. 4E, Supplementary Table 3). These genes were enriched within pyruvate metabolism, lipid metabolism and pentose and glucuronate interconversion pathways (Fig. 4F, Supplementary Table 3), suggesting that knockdown of *Wwox* in the presence of Aβ_42_ may alter the metabolome further via transcriptional regulation.

**Figure 4:**
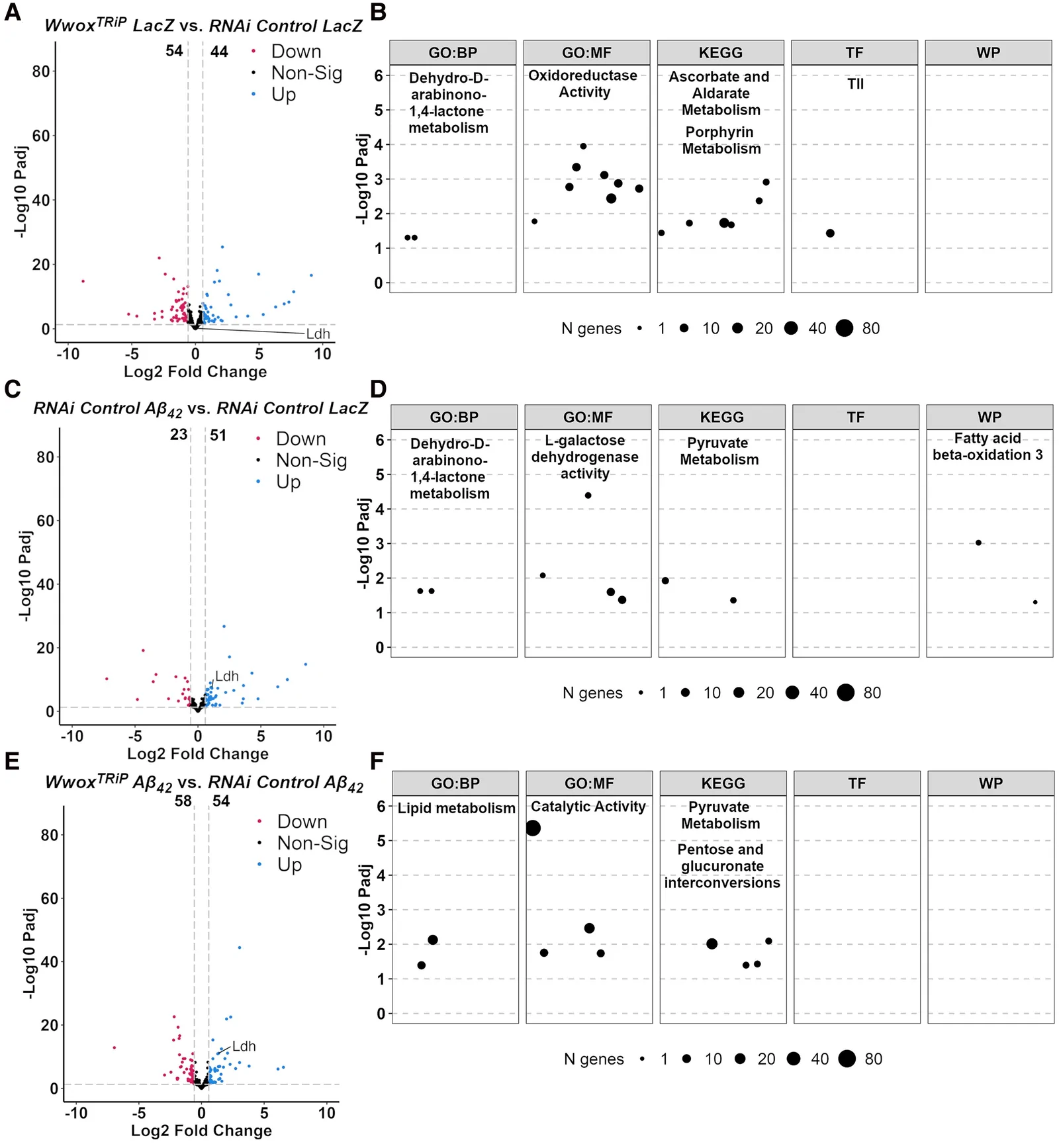
**Neuronal *Wwox* knockdown in the presence of Aβ_42_ vastly alters the metabolic transcriptome.** (**A**) Knockdown of *Wwox* in a control background differentially expressed (*P*_adj_ < 0.05, log_2_ fold-change threshold of ±0.58) 98 genes, 54 down (red) and 44 up (blue). (**B**) Pathway analysis revealed these were enriched in within metabolic pathways. (**C**) Volcano plot summarizing the significant up (51) and downregulated (23) genes after Aβ_42_ expression. (**D**) These were enriched within pyruvate and fatty-acid metabolism pathways. (**E**) Knockdown of *Wwox* in the presence of Aβ_42_ differentially expressed 112 genes (58 down, 54 up), (**F**) enriched within lipid and pyruvate metabolism terms. Dotted lines on volcano plots (panels A, C and E) represent log_2_ fold-change threshold of ±0.58. Overrepresentation analysis (panels B, D and F) was performed using g:Profiler, size of datapoints represents the number of genes identified in within the term. Abbreviations represent unique databases which were used for overrepresentation analysis GO:BP Gene ontology biological processes, GO:MF Gene ontology molecular functions, KEGG Kyoto Encyclopedia of Genes and Genomes, TF TRANSFAC, WP Wiki pathways. Transcriptomics was performed on *N* = 4 replicates of *n* = 10 female heads per genotype. Differential expression was performed using DEseq2, over representation pathway analysis was performed using g:Profiler. Non-sig = non-significant.

### *Wwox* knockdown in the presence of Aβ_42_ increases lactate concentrations via Atf4

Since various metabolic pathways were identified by our transcriptomic analysis, we performed HILC-based metabolomics to assess the effect of overexpression of Aβ_42_ and or *Wwox* knockdown on metabolic profiles of the *Drosophila* head (*N* = 5 replicates of *n* = 10 pooled heads). Of 2355 LC-MS peaks, 85 compounds mapped to known standards by m/z and retention time. Neuronal knockdown of *Wwox* differentially expressed (*P*_adj_ < 0.1) eight standard-mapped metabolites, namely, reduction of L-lysine (two peaks, *F* = 50.176 and F = 63.950 both *P*_adj_ < 0.0001), orotate (*F* = 18.036, *P*_adj_ = 0.0046) and 4-trimethylammoniobutanoate (*F* = 10.659, *P*_adj_ = 0.0263) and upregulation of D-glucuronate (*F* = 15.385, *P*_adj_ = 0.0069), 2-hydroxyglutarate (*F* = 10.672, *P*_adj_ = 0.0263), L-lactate (*F* = 7.524, *P*_adj_ = 0.0636) and itaconate (*F* = 6.310, *P*_adj_ = 0.0885, Fig. 5A, Supplementary Table 4). Aβ_42_ expression upregulated two standard-matched metabolites: 2-hydroxyglutarate (*F* = 49.551, *P*_adj_ = 0.0006) and L-lactate (*F* = 27.846, *P*_adj_ = 0.0050, Fig. 5B, Supplementary Table 4). Both lactate and 2-hydroxyglutarate are generated from pyruvate and α-ketoglutarate, respectively, by Ldh.^26,42^ This is consistent with our transcriptomics analysis, which revealed a 1.19 log_2_ fold-change upregulation of *Ldh* after overexpression of Aβ_42_ (Fig. 4C, Supplementary Table 3). Crucially, the interaction between neuronal *Wwox* knockdown and Aβ_42_ overexpression differentially expressed just one standard-mapped metabolite, L-lactate (*F* = 23.606, *P*_adj_ = 0.0174, 1.30 log_2_ fold-change Fig. 5C and D, Supplementary Fig. 5A, Supplementary Table 4). L-Lactate increased in the response to *Wwox* knockdown, only when Aβ_42_ was also present (Fig. 5C and Supplementary Fig. S5A), consistent with the 1.29 log_2_ fold-change increase in *Ldh* transcripts compared with Aβ_42_ overexpression alone in our transcriptomics experiment (Fig. 4E, Supplementary Table 3). In addition to this increase in *Ldh* and lactate, we observed corresponding transcriptional decreases in the pyruvate metabolism pathway—*Pepck2*, *CG11052* and *Adh—*further supporting the role of pyruvate metabolism in the phenotypes observed (Fig. 5D). An increase in *Ldh* and lactate suggests a Warburg effect-like shift towards glycolytic energy production. Interestingly however, we observed no differences in ATP levels in the brain as a consequence of *Wwox* knockdown (*F* = 0.3376, *P* = 0.5720), Aβ_42_ overexpression (*F* = 0.3811, *P* = 0.5486) or their interaction (*F* = 2.206, *P* = 0.1633, Supplementary Fig. 5B, *N* = 4), suggesting that the observed shift towards glycolytic metabolism is sufficient to maintain energy supply and may be a protective response.^43^

**Figure 5:**
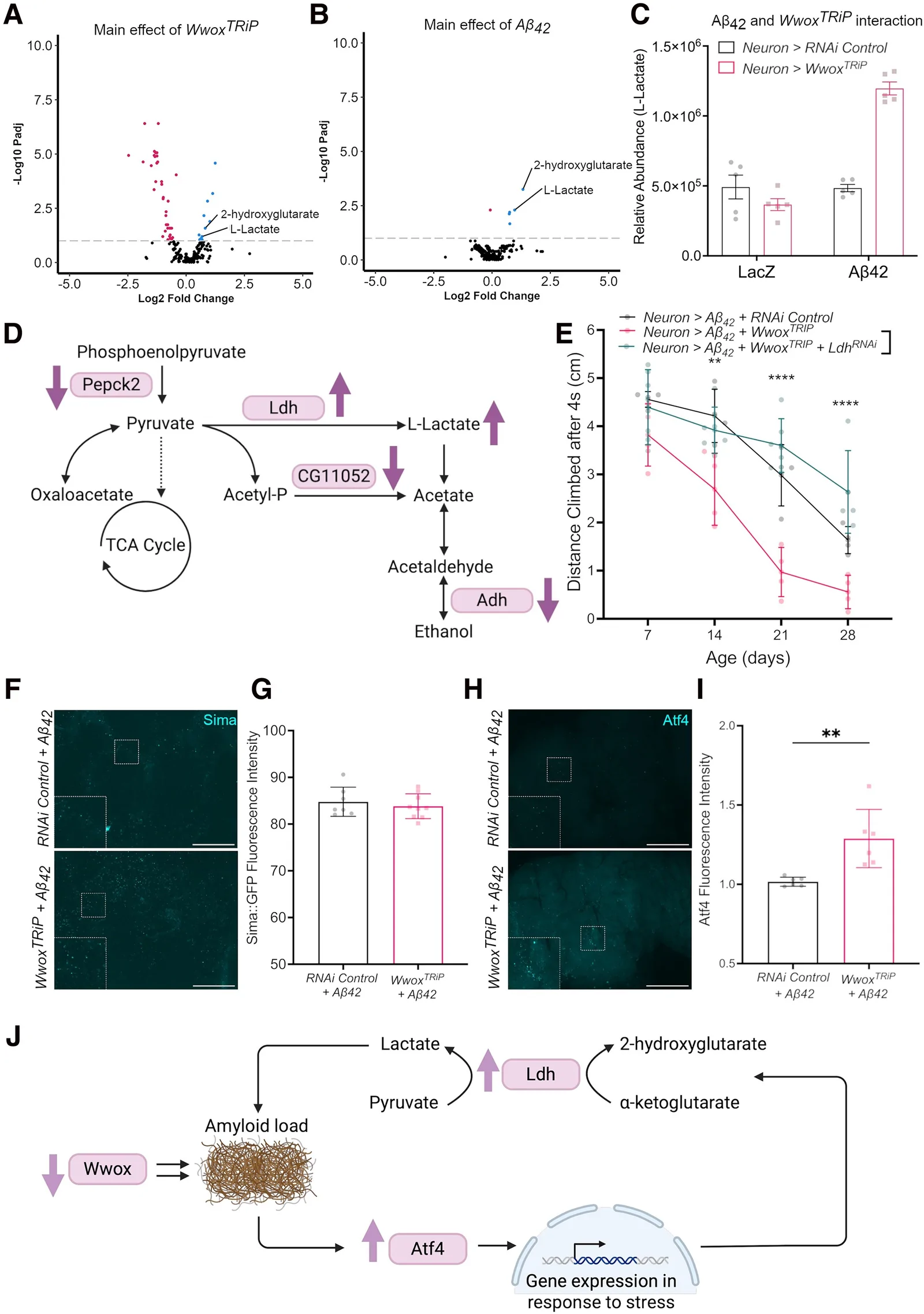
**Neuronal *Wwox* knockdown in the presence of Aβ_42_ overexpression increases L-lactate levels via increased lactate dehydrogenase expression.** (**A**) Volcano plots summarizing the significant (*P*_adj_ < 0.1) up- and downregulated metabolites for the main effect of *Wwox* knockdown or (**B**) the main effect of Aβ_42_ (day 14). (**C**) Metabolite counts for L-lactate in each condition reveals a large increase, after knockdown of *Wwox* in the presence of Aβ_42_ (*N* = 5 replicates of *n* = 10 pooled heads, mean ± SEM). (**D**) Schematic of changes in pyruvate metabolism revealed by the statistical interaction between *Wwox* knockdown and Aβ_42_ overexpression from omic analyses. Differentially expressed transcripts and metabolites are depicted with arrows showing their direction of change, transcripts are represented in boxes. Created in BioRender. Lucas-Clarke, H. (2026) https://BioRender.com/fwthumj. (**E**) Knockdown of lactate dehydrogenase (*Ldh*) rescued startled induced locomotor deficits in *Wwox* knockdown (+Aβ_42_) flies at 14-, 21- and 28 days (*N* = 4–6 of *n* = 5–10 flies). (**F**) Representative images of brains expressing the endogenously tagged Sima:GFP reporter in day 14 flies. (**G**) Quantification revealed no differences in sima levels (*N* = 7–9 brains). (**H**) Atf4 immunocytochemistry in day 14 fly brains, (**I**) demonstrated an upregulation of Atf4 in the brain after *Wwox* knockdown in the presence of Aβ_42_ (*N* = 6 brains). (**J**) Schematic representation of the effect of *Wwox* knockdown on pyruvate metabolism and Aβ_42_ aggregation via Atf4. Created in BioRender. Lucas-Clarke, H. (2026) https://BioRender.com/4ljuoko. Statistics = Two-way ANOVA followed by multiple testing correction with 10% FDR (panels A–C), two-way ANOVA mixed effect model (Šídák's multiple comparisons correction) (panel **E**), two-tailed unpaired *t*-test (panel **G**) and Mann-Whitney Test (panel I). *P*/*P*_adj_ * < 0.05, ** < 0.01, *** < 0.001, **** < 0.0001. Mean ± SD, unless otherwise stated. Scale bars = 100 µm. Dotted line in panels A–C represents *P*_adj_ = 0.1. All experiments were performed on females. Datapoints represent *N*: independent replicates of pooled heads (panel **C**), independent vials (panel **E**) and independent brains (panels **G**, **J**).

Given the consistent changes in lactate/*Ldh* across our omic analyses, previous literature implicating *Ldh* in amyloid toxicity,^43^ and our results suggesting that *Wwox* modulation may be an upstream regulator of this toxicity, we next investigated the physiological consequences of elevated *Ldh* and lactate seen in Aβ_42_-transgenic flies with reduced *Wwox* expression. We utilized RNAi to mediate combined downregulation of both *Ldh* and *Wwox* in neurons expressing Aβ_42_ (Supplementary Fig. 5C) and assessed startle-induced locomotion. Multiple comparisons testing revealed that *Ldh* knockdown rescued climbing deficits seen in Aβ_42_-transgenic flies with downregulated *Wwox* (at 14-, 21- and 28-days *t* = 3.182 *P*_adj_ = 0.0081, *t* = 6.832 *P*_adj_ < 0.0001 and *t* = 5.198 *P*_adj_ < 0.0001, respectively, Fig. 5E, *N* = 4–6 vials). These results suggest that *Wwox* knockdown in the presence of Aβ_42_ elevates lactate levels via neuronal *Ldh*, which is sufficient to exacerbate locomotor defects.

Both HIF1α and Atf4 are transcription factors known to promote *Ldh* expression. We investigated the levels of these proteins upon *Wwox* knockdown to further understand *Ldh* regulation in our model. No changes in endogenously tagged sima:GFP (HIF1α homolog) levels were observed in the adult (day 14) fly brains after neuronal *Wwox* knockdown in Aβ_42_ overexpressing flies (*t* = 0.6623, *P* = 0.5185, Fig. 5F and G, *N* = 7–9). However, immunocytochemistry revealed a significant upregulation of Atf4 levels in Aβ_42_ flies upon *Wwox* knockdown (*U* = 0 *P* = 0.0022, Fig. 5H and I, *N* = 6), which were not observed in LacZ-expressing *Wwox* knockdown flies (Supplementary Fig. 5D–E). Consistent with the notion that Atf4 is minimally expressed at rest in the adult brain, but upregulated upon stress.^26^ Since Atf4 activation occurs downstream of protein kinase R (PKR)-like endoplasmic reticulum kinase (PERK) through the ISR and UPR,^44^ we assessed if the Ire1-Xbp1 branch of the UPR was affected, by measuring Ire1-mediated *Xbp1* splicing with a GFP-based reporter expressed in neurons.^45^ However, we observed no differences in neuronal spliced *Xbp1* because of *Wwox* knockdown in Aβ_42_ flies (*t* = 1.365, *P* = 0.1954, Supplementary Fig. 5F and G, *N* = 6–9). These data suggest that neuronal *Wwox* knockdown specifically upregulates the PERK-Atf4 branch of the ISR/UPR in Aβ_42_ flies and this could be the mechanism via which *Ldh* and lactate are upregulated, leading to exacerbated Aβ_42_-induced phenotypes (Fig. 5J).

### Upregulation of neuronal *Wwox* rescues Aβ_42_ related pathology

Having described the detrimental effect of *Wwox* knockdown on Aβ_42_-induced phenotypes, we wanted to explore whether *Wwox* upregulation could also affect Aβ_42_ toxicity. We employed CRISPRa upregulation of *Wwox* by expressing *UAS-dCas9-VPR* and tandem gRNAs targeting the *Wwox* transcriptional start site (*Wwox*^gRNA^) or non-targeting (control^gRNA^), under pan-neuronal *elav-GAL4* control. This was sufficient to achieve 2-fold increase in *Wwox* mRNA levels in the fly head (Supplementary Fig. 1), whilst not affecting the mRNA levels of Aβ_42_ when it was overexpressed (Supplementary Fig. 3A). In the absence of Aβ_42_, *Wwox* upregulation had no significant effect on survival (median = 60 versus 58 days,  c^2^ = 2.156, *P* = 0.1420, Supplementary Fig. 6A, *N* = 76–86) or startle-induced locomotion (*F* = 0.02434, *P* = 0.8770, Supplementary Fig. 6B, *N* = 3–6 vials). However, in the presence of neuronal Aβ_42_, upregulation of *Wwox* significantly improved lifespan (median = 47.5 versus 29 days, χ^2^ = 104.5, *P* < 0.0001, Fig. 6A, *N* = 78–91) and startle-induced locomotion (*F* = 42.33, *P* < 0.0001) at days 14 and 21 (*t* = 5.669 and *t* = 6.169 both *P*_adj_ < 0.0001, Fig. 6B, *N* = 3–7 vials). We observed a similar rescue of lifespan (median = 41 versus 51 days, χ^2^ = 9.493 *P* = 0.0021, Supplementary Fig. 6C, *N* = 85–106) when overexpressing a *UAS*-cDNA construct of human *WWOX* (*hWWOX*) in neurons. Since different posttranslational modifications to WWOX have been previously implicated in Tau pathology,^46,49^ we generated phosphomimic and phosphomutant cDNA variants of *hWWOX* at residues Ser14 and Tyr33. These variants performed similarly to wildtype *hWWOX* in their rescue of Aβ_42_ induced locomotor function (Supplementary Fig. 6D). Notably, *dWwox* cDNA overexpression provided a better rescue of locomotor ability than *hWWOX* and *hWWOX* Ser14 phosphomutant (Supplementary Fig. 6D).

**Figure 6:**
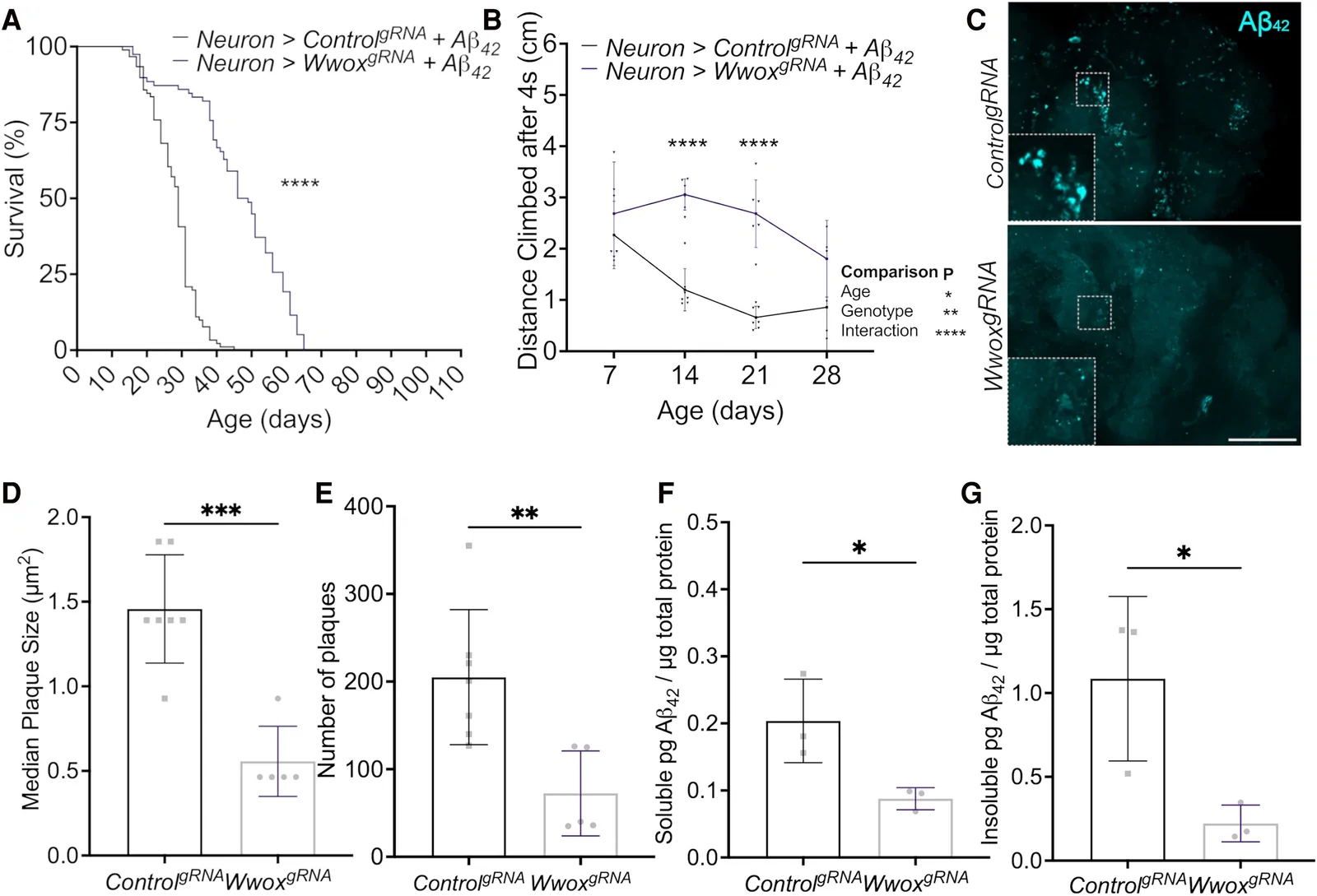
**Neuronal upregulation of *Wwox* improves longevity, behaviour, and reduces amyloid load.** (**A**) Upregulation of *Wwox* in neurons expressing Aβ_42_ significantly improved lifespan (*N* = 78–91 individual flies), (**B**) and distance climbed after 4 seconds (s) in a startle induced locomotion assay locomotion (*N* = 3–7 vials of *n* = 5–10 flies) at days 14 and 21. (**C**) Representative images of Aβ_42_ staining (cyan) in *Drosophila* brains (day 14). (**D**) Quantification revealed a significant decrease in median plaque size and (**E**) number (*N* = 5–7 brains). Using the MSD assay for amyloid we revealed decreased (**F**) soluble and (**G**) insoluble Aβ_42_ (*N* = 3 replicates of *n* = 25 heads). *P*/*P*_adj_ * < 0.05, ** < 0.01, *** < 0.001, **** < 0.0001. Mean ± SD. Scale bar = 100 µm. Statistics = Mantel-Cox tests (panel **A**), two-way ANOVA mixed effect model (Šídák's multiple comparisons correction) (panel **B**) and two-tailed unpaired *t*-tests (panels **D–G**). All experiments were performed on females. Datapoints represent *N*: independent vials (panel **B**), independent brains (panels **D** and **E**) and independent replicates of pooled heads (panel **F** and **G**).

Overexpression of *Drosophila* or *hWWOX* cDNA in glial cells whilst simultaneously overexpressing Aβ_42_ in neurons had no effect on survival (χ^2^ = 0.0644 *P*_adj_ = 0.7997 and χ^2^ = 3.768 *P*_adj_ = 0.1045 respectively, Supplementary Fig. 6E, *N* = 84–154) or locomotion (F = 0.4914, *P* = 0.6654, Supplementary Fig. 6F, *N* = 4–7 vials) in comparison to controls, further indicating the importance of neuronal, compared with glial *Wwox*, in the modulation of neuron-derived Aβ_42_ toxicity. In the brains of flies with *Wwox* upregulation in neurons, we found a dramatic reduction in the median size (*t* = 5.485, *P* = 0.0003, Fig. 6C and D) and number (*t* = 3.374, *P* = 0.0071, Fig. 6C and E, *N* = 5–7) of Aβ_42_ deposits with confocal microscopy. Similarly, by MSD assay, we found reduced total Aβ_42_ (*t* = 3.252, *P* = 0.0313, Supplementary Fig. 6G) in both soluble (*t* = 3.114, *P* = 0.0357, Fig. 6F) and insoluble (*t* = 2.975, *P* = 0.0409, Fig. 6G, *N* = 3) head lysate fractions of day 14 flies, without altering the ratio of insoluble/soluble Aβ_42_ (*t* = 1.334, *P* = 0.2530, Supplementary Fig. 6H, *N* = 3). This suggests that increasing neuronal *Wwox* can protect against Aβ_42_ associated pathology by lowering total amyloid load.

### Neuronal upregulation of *Wwox* reverses the transcriptional profile induced by Aβ_42_

To understand how neuronal upregulation of *Wwox* may provide protection against Aβ_42_ and since *Wwox* knockdown substantially altered the transcriptomic profile of Aβ_42_ flies, we performed bulk transcriptomics on the heads of flies with *Wwox* upregulation in neurons (*N* = 3–4 replicates of *n* = 10 pooled heads). PCA only partially distinguished the effects of *Wwox* overexpression and Aβ_42_ with PC1 explaining 29% and PC2 16% of variance (Supplementary Fig. 7A). This lack of separation between control and overexpression in the LacZ background was reflected by no upregulated genes and downregulation of only six genes (Fig. 7A, Supplementary Table 5)—these downregulated genes were enriched for fatty acid metabolism terms (Supplementary Fig. 7B, Supplementary Table 5). Overexpression of Aβ_42_ significantly downregulated 51 genes and upregulated 28 (Fig. 7B, Supplementary Table 5), of which *Ldh* was the second most significantly upregulated gene with a 2.85 log_2_ fold-change compared with control. Over representation analysis revealed dysregulation of translation repression, membrane transport and oxidative phosphorylation (Supplementary Fig. 7C, Supplementary Table 5). There was a significant overlap between the differentially expressed genes identified upon Aβ_42_ expression in the knockdown and overexpression experiments, including *Ldh*, *Hsc70-3*, *CG11842*, *GNBP-like3*, *BomBc3* and *Oat,* thus implicating ER stress response, Toll receptor activation and energy metabolism as reproducible changes on the transcriptional level in this amyloid model (Supplementary Fig. 7D, OR = 28.52, *P* < 0.0001, Fisher Exact Test).

**Figure 7:**
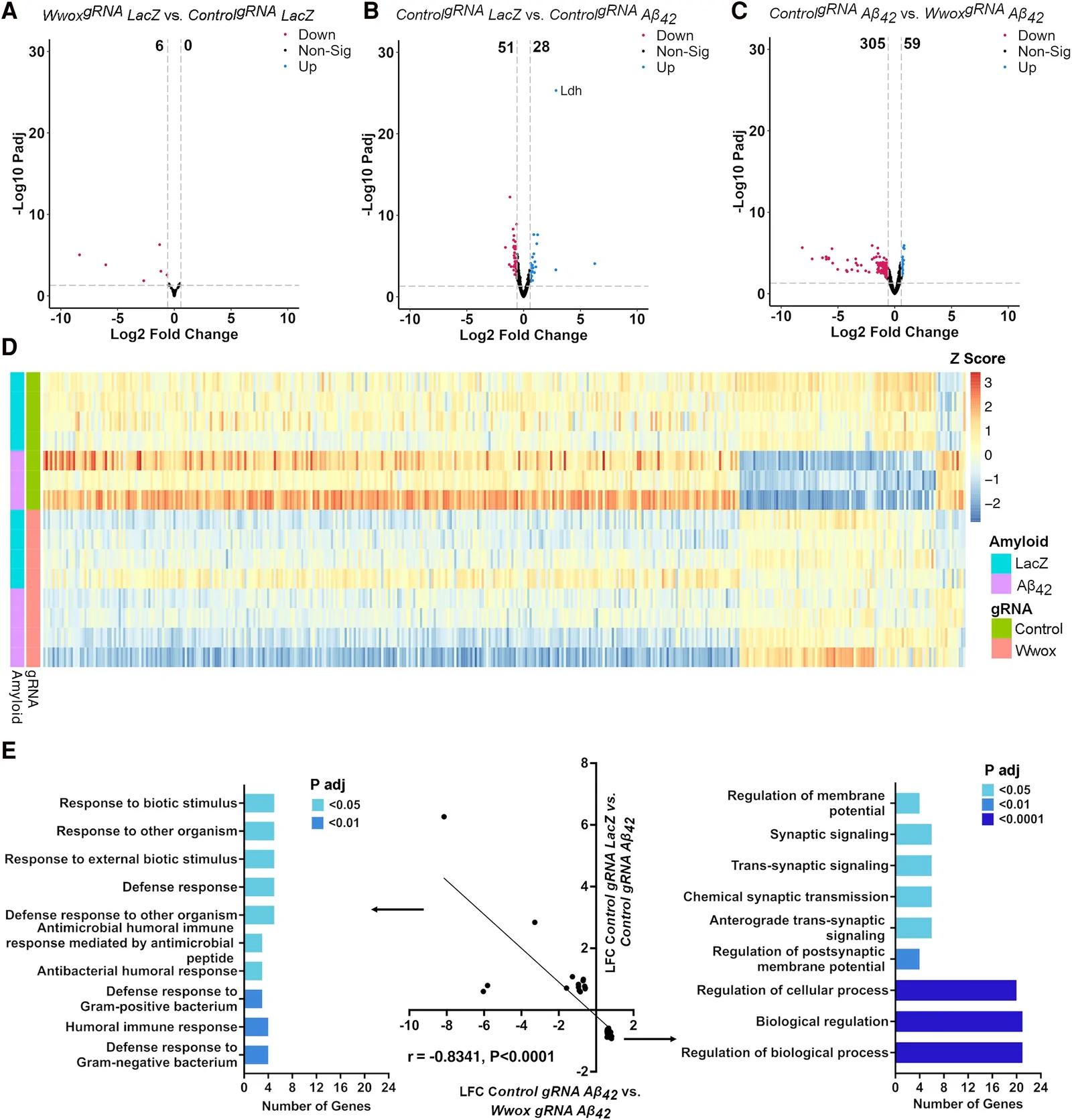
**Neuronal overexpression of *Wwox* reverses the metabolic, immune and synaptic transcriptomic reprogramming caused by Aβ_42_ expression.** (**A**) Volcano plot for the effect of *Wwox* overexpression in a control background reveals dysregulation of six genes (down), (**B**) overexpression of Aβ_42_ downregulated 51 genes and upregulated 28, and (**C**) overexpression of *Wwox* in the presence of Aβ_42_ differentially expressed 364 genes (305 down, 59 up). (**D**) Heatmap of *z*-scores of all the uniquely differentially expressed genes and their expression in each condition reveals a clear reversal of the amyloid signature with *Wwox* overexpression. (**E**) 40 genes were commonly dysregulated between Aβ_42_ expression alone and Aβ_42_ expression with *Wwox* overexpression. The log_2_ fold-change of each of these genes were significantly negatively correlated suggesting a reversal of the transcriptional profile. Those genes that were upregulated by Aβ_42_ and subsequently downregulated with *Wwox* overexpression were enriched within immune pathways (left) whereas genes that change in the opposing direction were enriched with synaptic transmission. Datapoints in panel **E** represent each of these individual 40 genes. Dotted lines on volcano plots (panels **A–C**) represent log_2_ fold-change threshold of ±0.58 and *P*_adj_ threshold of 0.05. *N* = 4 replicates of *n* = 10 pooled heads. Statistics = Differential expression was performed using DEseq2 (panels **A–C**), pathway analysis was performed using g:Profiler (panel **E**), *z*-scores (panel **D**), Pearson correlation coefficient (panel **E**). All experiments were performed on day 14 females. LFC = log_2_ fold-change, Non-sig = non-significant.

Strikingly, upregulation of neuronal *Wwox* in the presence of Aβ_42_, downregulated 305 genes and upregulated 59 (Fig. 7C, Supplementary Table 5). Overrepresentation analysis revealed metabolic and immune enrichments, including a dysregulation of cysteine and methionine metabolism lead largely by these downregulated genes (Supplementary Fig. 7E, Supplementary Table 5). Markedly, the expression of the 404 unique genes differentially expressed appear to shift in directional change, implying *Wwox* overexpression reverses amyloid related transcriptional signatures (Fig. 7D). Specifically, 40 of these genes were common between the analyses of Aβ_42_ versus LacZ and *Wwox*^gRNA^ Aβ_42_ versus control^gRNA^ Aβ_42_ (Supplementary Table 6), the log_2_ fold-changes of these genes were significantly negatively correlated (*r* = −0.8341, *P* < 0.0001, Fig. 7E). Notably, every gene differentially expressed in Aβ_42_ versus LacZ comparison was differentially expressed in the opposing direction by the *Wwox*^gRNA^ Aβ_42_ versus control^gRNA^ Aβ_42_ comparison. Overrepresentation analysis revealed that those genes upregulated by Aβ_42_ and downregulated by *Wwox* overexpression were enriched within immune response terms, whereas genes with the opposing change were enriched for synaptic terms (Fig. 7E). Taken together, these results suggest that upregulation of *Wwox* reverses the transcriptional reprogramming of metabolic, immune and synaptic pathways caused by Aβ_42_ upregulation.

### Neuronal upregulation of *Wwox* reverses transcriptional pathways dysregulated by Aβ_42_

To understand how this transcriptional reprogramming may impact neuronal metabolism of these flies, we performed unbiased metabolomic profiling on flies expressing Aβ_42_, overexpressing *Wwox*, or overexpressing *Wwox* in the presence of Aβ_42_ (*N* = 5 replicates of *n* = 10 heads). Of 2414 peaks, 86 compounds were exact matches to known standards by m/z and retention time. Expression of Aβ_42_ differentially altered four standard-matched metabolites (*P*_adj_ < 0.1), including a reduction in sn-glycero-3-phosphocholine (*F* = 35.42, *P*_adj_ = 0.0006) and an increase in betaine (*F* = 8.164, *P*_adj_ = 0.0667), 2-hydroxyglutarate (*F* = 47.94, *P*_adj_ = 0.0001) and citrate (*F* = 8.197, *P*_adj_ = 0.0667, Fig. 8A, Supplementary Table 7). This increase in 2-hydroxyglutarate is consistent with our previous finding of a 2.85 log_2_ fold-change increase in *Ldh* transcripts in Aβ_42_ expressing flies compared with control—the second most significant differentially expressed gene in this comparison (Supplementary Table 5). Neuronal *Wwox* overexpression downregulated five standard-matched metabolites: L-leucine (*F* = 9.949, *P*_adj_ = 0.0545), sn-glycero-3-phosphocholine (*F* = 9.311, *P*_adj_ = 0.0667), L-methionine (*F* = 22.15 *P*_adj_ = 0.0047 and F = 17.38 *P*_adj_ = 0.0091) and a suspected isomer of methyl-L-histidine (*N*-pi) (*F* = 9.93 *P*_adj_ = 0.0545, Fig. 8B, Supplementary Table 7). However, the interaction between *Wwox* overexpression and Aβ_42_ revealed just the L-methionine metabolite (*F* = 19.79 *P*_adj_ = 0.0073 and *F* = 14.92 *P*_adj_ = 0.0133, Fig. 8C, Supplementary Table 7). L-methionine levels were decreased after *Wwox* overexpression in the control background but were unaltered by overexpression of *Wwox* in the presence of Aβ_42_ (Fig. 8C, Supplementary Fig. 8A and B). Surprisingly, the reduction of amyloid load and improvement in lifespan and locomotion of these flies therefore occurred without restoring *Ldh* transcript or 2-hydroxyglutarate levels but instead caused a reduction in transcripts encoding numerous enzymes within the cysteine and methionine metabolism pathway (Fig. 8D). Reduced L-methionine levels may represent a protective response to Aβ_42-_induced stress, while *Wwox* overexpression appears to regulate the same pathway transcriptionally to maintain these low L-methionine levels.

**Figure 8:**
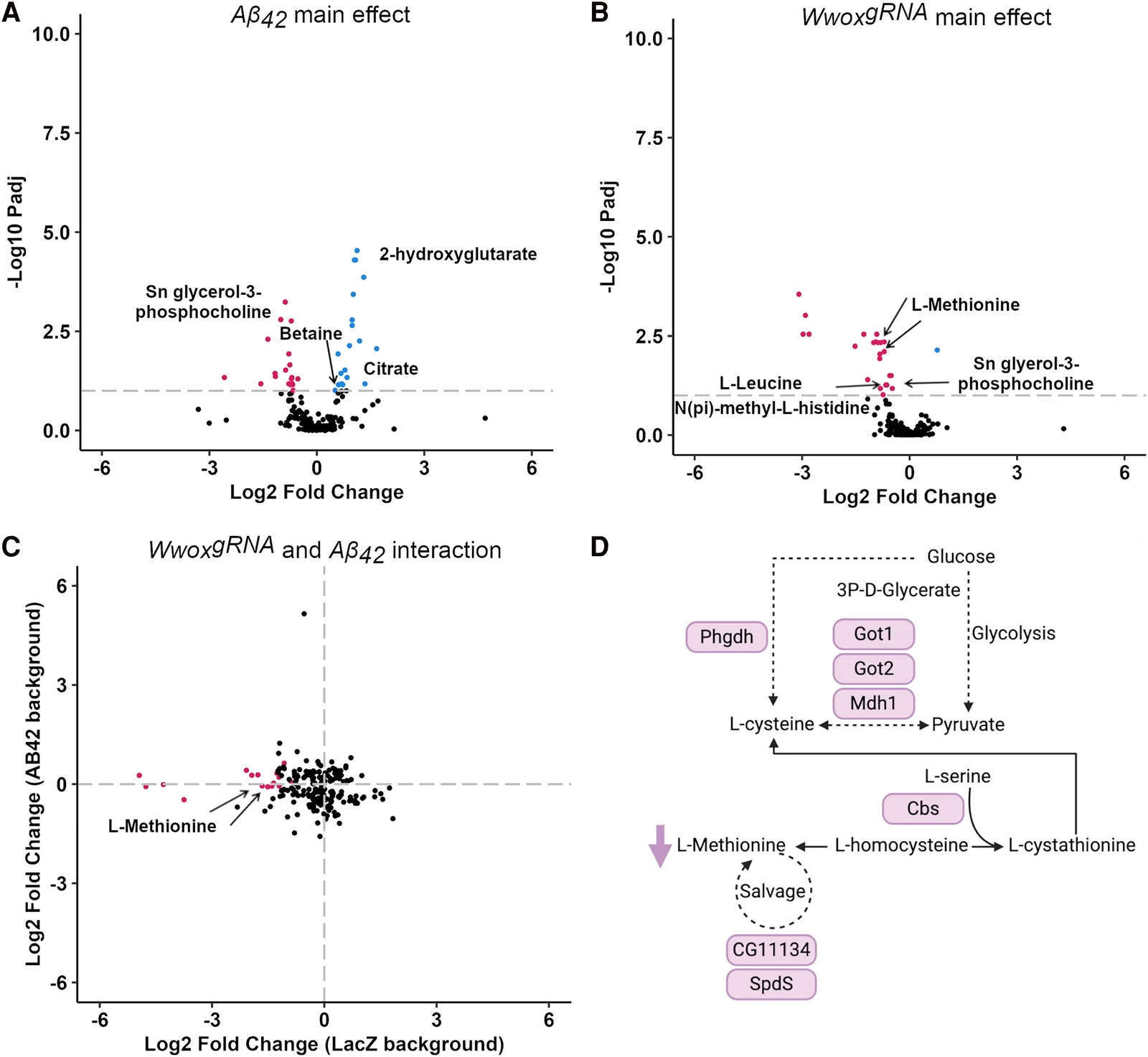
**Neuronal overexpression of *Wwox* alters L-methionine levels.** Volcano plots (**A**) illustrating the differentially expressed metabolites due to the main effects of Aβ_42_ expression or (**B**) *Wwox* overexpression. Labelled metabolites significantly altered after FDR correction (*P*_adj_ < 0.1) and standard matched. (**C**) Log_2_ fold-changes were plotted for the effect of *Wwox* overexpression in a LacZ background or Aβ_42_ background, revealing differing effects on L-methionine levels. (**D**) Schematic of enzymes encoded by downregulated transcripts (pink boxes) within the cysteine and methionine metabolism KEGG pathway revealed in our analysis of *Wwox* overexpression in the presence of Aβ_42_. L-methionine metabolites were reduced in the Aβ_42_ model (pink arrow) and remain low when *Wwox* is overexpressed in the presence of Aβ_42_ (interaction effect). Created in BioRender. Lucas-Clarke, H. (2026) https://BioRender.com/zz1zyt8. *N* = 5 replicates of *n* = 10 pooled day 14 female heads. Statistics = two-way ANOVA followed by multiple testing correction with 10% FDR.

## Discussion

In this study we investigated the multifaceted, cell-type specific effects of *Wwox* manipulation in the *Drosophila* brain, in combination with the effects of ageing and human Aβ_42_ overexpression. Like mammals,^40^  *Drosophila Wwox* is expressed throughout the brain in both neuronal and glial cell types. Using RNAi, we reduced *Wwox* levels in these cell types to recapitulate the subtle dysregulation of expression observed in LOAD.^3,5^ Whilst startle-induced locomotion was unaffected by *Wwox* RNAi in neurons or glia, knockdown in either cell type reduced lifespan, suggesting its important role in both neuronal and glial health. However, it is important to note that startle-induced locomotor phenotypes may emerge later in the flies’ lifespan as reduction in *Wwox* caused more subtle changes in general activity captured at baseline in the longitudinal DAM experiment. Additionally, *Wwox* knockdown in either cell type affected sleep homeostasis, decreasing activity and increasing sleep, without disrupting circadian rhythmicity. These results suggest an involvement of *WWOX* in both age-related sleep decline and abnormalities seen in early-stage LOAD.^50,51^ Supporting our findings, a recent study also reported sleep defects in *Wwox* knockout *Drosophila* whilst also demonstrating a genome wide association between SNPs at the *WWOX* locus and self-aware sleep duration.^47^ Furthermore, a genome wide analysis for gene-sleep length interactions in the context of hypertension identified a SNP mapping to *WWOX* associated with short sleep (rs542745170).^52^ Taken together these results suggests a role of WWOX modulating sleep duration across diseases.

*Wwox* knockdown in the Aβ_42_ model of Ad exacerbated neurotoxicity, leading to increase Aβ_42_ load, impaired locomotor function and reduced lifespan. However, *Wwox* knockdown in glia had no effect on the toxicity of neuron-derived Aβ_42_, in the context of startle-induced locomotor function, suggesting that neuronal *Wwox* plays a cell-autonomous protective role against Aβ_42_-mediated toxicity. Metabolic exploration of Aβ_42_ expression revealed an increase of 2-hydroxyglutarate and L-lactate—the products of Ldh conversion of α-ketoglutarate and pyruvate, respectively.^26,42^ Consistently we found *Ldh* transcripts significantly increased with Aβ_42_ expression. This enzymatic upregulation could explain previous reports of increased lactate levels in Aβ_42_-expressing *Drosophila* and Ad patients.^53,54^ Keeping Ldh levels controlled in the brain could be key to maintaining normal metabolic homeostasis and *Ldh* overexpression has been found to shorten lifespan, disrupt circadian rhythms and induce neurodegeneration.^55,56^ Interestingly, *Ldh* knockdown has also been shown to cause detrimental effects in fly Aβ_42_ models, suggesting that *Ldh* expression and lactate levels need to be tightly controlled.^43,57^ Here we identified that *Wwox* knockdown combined with Aβ_42_ expression further elevated L-lactate levels, likely mediated by the further increase in *Ldh* expression observed in our transcriptomics data. Taken together, these data provide evidence that some genetic risk loci, like *WWOX*, may be acting upstream of transcriptional regulation of the metabolome to control pathology, thereby influencing disease progression.

The transcription factors Atf4 and HIF1α are both known to increase transcription of *Ldh*.^15,43^ While WWOX has been shown to inhibit HIF1α,^9^ our data suggest that Atf4, rather than the HIF1α orthologue sima, is primarily responsible for the observed *Ldh* upregulation in this fly model. Indeed, Atf4-dependent, sima-independent upregulation of *Ldh* has been previously reported in Aβ_42_-expressing *Drosophila*,^43^ further supporting these results. Atf4 is upregulated by stimulation of the PERK arm of the ISR/UPR pathway via phosphorylation of eif2α which supresses global translation but specifically promotes translation of specific stress response factors, including Atf4. Whilst we did not determine whether WWOX is activating this pathway directly in this study, previous research has shown that WWOX can interact in proximity with PERK and could be directly affecting its activation.^58^ Furthermore, WWOX has also been shown to directly bind GCN1,^56^ an activator of Eukaryotic Translation Initiation Factor 2 Alpha Kinase 4 (GCN2)—a kinase known to phosphorylate eif2α.^59^ GCN2 depletion has previously been shown to be detrimental in the 5xFAD mouse model of Aβ_42_ toxicity, via enhanced PERK activation and is elevation.^60^ WWOX regulation of GCN2 could thus also explain the elevated Atf4 and aggravation of Aβ_42_ toxicity in our *Wwox* knockdown model. Interestingly, this pathway is not linear—previous research has shown that hippocampal neurons exposed to Aβ_42_ oligomers can increase transport of *Atf4* mRNA in axons, where it is locally translated before retrograde transport to the soma, leading to prolonged CHOP expression and CHOP-dependant cell-death.^61^ Whilst no *Drosophila* CHOP orthologue has been identified, prolonged PERK/Atf4 signalling has been shown to cause cell death in *Drosophila* overexpressing Presenilin, which encodes the catalytic subunit of Aβ_42_ liberating γ-secretase.^62^ Our findings suggest that the combined effect of *Wwox* knockdown and Aβ_42_ leads to chronic ISR/UPR activation, characterized by elevated Atf4, *Ldh* and lactate, above that of Aβ_42_ overexpression alone. Previous work has demonstrated that Aβ expression induces *Xbp1* splicing and activation of the ER stress response, and that the active isoform Xbp1 exerts a neuroprotective effect against Aβ toxicity.^63^ However, we do not find that this pathway is linked to Wwox regulation of the amyloid phenotypes.

Notably, we found that reducing *Ldh* levels in neurons rescued *Wwox* knockdown-induced locomotor deficits in the presence of Aβ_42,_ suggesting that elevated neuron-derived lactate levels, downstream of Atf4 signalling, could be driving the detrimental effects of *Wwox* knockdown in Aβ_42_ overexpressing flies. Lactate has been shown to be detrimental in amyloid models previously, for instance, lactic acid accumulation may increase APP-Grp78 binding, subsequent amyloidogenic cleavage and UPR disinhibition.^64^ Additionally, aerobic glycolysis has been suggested to directly modulate Aβ_42_ accumulation,^65,66^ implying a direct link between lactate and amyloid through *Ldh*. Accordingly, *Ldh* knockdown provided a rescue in our model. Taken together these results place *Wwox* upstream of Atf4, *Ldh* and Lactate, and suggest it is this increase in lactate that may increase amyloid toxicity. However, the rescue we witnessed was partial, suggesting that other alternative pathways may also be contributing to the phenotypes. The change observed in 2-hydroxyglutarate levels is of particular interest and is only partially controlled by *Ldh*. 2-hydroxyglutarate has been extensively explored in the context of cancer and enantiomers can be generated via action of numerous enzymes including isocitrate dehydrogenase, malate dehydrogenase and phosphoglycerate dehydrogenase.^67^ Therefore, further research is needed to understand the contribution of several converging metabolic pathways in the context of amyloid pathology.

In addition to identifying deleterious effects of *Wwox* knockdown, recapitulating human risk, we found that upregulation of *Wwox* remarkably reduced amyloid load, rescued lifespan and locomotion of Aβ_42_ overexpressing flies. Surprisingly however, omics analyses show this rescue occurs without a reduction in *Ldh* or 2-hydroxyglutarate, suggesting a different mechanism of protection. We observed a dramatic reversal of transcriptional changes after *Wwox* overexpression in the presence of amyloid, identifying a downregulation of metabolic and inflammatory related genes. The reduction in L-methionine observed in the Aβ_42_ model brain may reflect a protective adaptation to Aβ_42_-induced stress, whereas *Wwox* overexpression appears to transcriptionally regulate this pathway to sustain lower L-methionine levels. This suggests that Wwox may play a critical role in maintaining metabolic brain homeostasis and regulating amino acid pathways under normal physiological conditions. L-methionine is an essential amino acid; however, it has been suggested that diets high in methionine can result in worsened memory,^68,69^ neurodegeneration and increased Aβ_42_ deposition in mouse models.^69^ Interestingly chronic L-methionine administration has also been shown to increase inflammatory cytokine release from microglial cells.^70^ Therefore, there is a potential that overexpressing neuronal *Wwox*, and thus altering neuronal metabolism, is conferring protection through glia—an area for future research. In this study we suggest differing mechanisms conferring vulnerability and protection in the context of amyloid pathology via modulation of *Wwox*. Our findings support the growing literature implicating WWOX in numerous metabolic pathways,^71^ many of which lead to the generation of acetyl-CoA from degradation of proteins, lipids or carbohydrates.^56^ Wwox can be seen as involved in broad metabolic reprogramming and fuel selection in neurons, with metabolomic analysis revealing alterations in glucose (lactate, 2-hydroxyglutarate), amino acid (L-lysine, 4-Trimethylammoniobutanoate, D-Glucuronate, L-methionine, methyl-L-histidine (*N*-pi), and L-leucine), lipid (sn-glycero-3-phosphocholine), and nucleotide metabolism (D-Glucuronate, orotate).

In conclusion, our study provides new insights into the multifaceted relationship between Aβ_42_ expression and *Wwox* levels in neurons. We demonstrate that *Wwox* plays a critical role in neuronal health and the modulation of Aβ_42_ toxicity. Additionally, we identified metabolic alterations associated with Aβ_42_ expression and *Wwox* knockdown. Excitingly, increasing *Wwox* expression in neurons caused a dramatic rescue of amyloid load. Future studies should explore the potential therapeutic implications of targeting these pathways for treatment of Ad.

## Supplemental material

Supplementary material is available at Brain Communications online.

## Supplementary Material

fcag385_Supplementary_Data

## Data Availability

Data analysis code is available at https://github.com/Fly-Cardiff. Raw reads and feature counts for the transcriptomics experiment can be accessed on Array Express, for both the knockdown (E-MTAB-14948) and overexpression (E-MTAB-14949) experiments. The metabolomics data have been deposited to MetaboLights repository with the study identifier MTBLS12344.

## References

[1] Bellenguez C, Grenier-Boley B, Lambert JC. Genetics of Alzheimer’s disease: Where we are, and where we are going. Curr Opin Neurobiol. 2020;61:40–48. 10.1016/j.conb.2019.11.024 31863938

[2] Kunkle BW, Grenier-Boley B, Sims R, et al Genetic meta-analysis of diagnosed Alzheimer’s disease identifies new risk loci and implicates Aβ, tau, immunity and lipid processing. Nat Genet. 2019;51(3):414–430. 10.1038/s41588-019-0358-2 PMC6463297 30820047

[3] Patel D, Zhang X, Farrell JJ, et al Cell-type-specific expression quantitative trait loci associated with Alzheimer disease in blood and brain tissue. Transl Psychiatry. 2021;11(1):250. 10.1038/s41398-021-01373-z PMC8079392 33907181

[4] Stevenson-Hoare J, Heslegrave A, Leonenko G, et al Plasma biomarkers and genetics in the diagnosis and prediction of Alzheimer’s disease. Brain. 2023;146(2):690–699. 10.1093/brain/awac128 PMC9924904 35383826

[5] Sze CI, Su M, Pugazhenthi S, et al Down-regulation of WW domain-containing oxidoreductase induces tau phosphorylation in vitro: A potential role in Alzheimer’s disease. J Biol Chem. 2004;279(29):30498–30506. 10.1074/jbc.M401399200 15126504

[6] Chang JY, Chang NS. WWOX dysfunction induces sequential aggregation of TRAPPC6AΔ, TIAF1, tau and amyloid β, and causes apoptosis. Cell Death Discov. 2015;1(1):15003. 10.1038/cddiscovery.2015.3 PMC4981022 27551439

[7] Sze CI, Kuo YM, Hsu LJ, et al A cascade of protein aggregation bombards mitochondria for neurodegeneration and apoptosis under WWOX deficiency. Cell Death Dis. 2015;6(9):e1881. 10.1038/cddis.2015.251 PMC4650446 26355344

[8] Wang HY, Juo LI, Lin YT, et al WW domain-containing oxidoreductase promotes neuronal differentiation via negative regulation of glycogen synthase kinase 3β. Cell Death Differ. 2012;19(6):1049–1059. 10.1038/cdd.2011.188 PMC3354054 22193544

[9] Abu-Remaileh M, Seewaldt VL, Aqeilan RI. WWOX loss activates aerobic glycolysis. Mol Cell Oncol. 2015;2(2):e965640. 10.4161/23723548.2014.965640 PMC4904998 27308416

[10] O’Keefe LV, Colella A, Dayan S, et al *Drosophila* orthologue of WWOX, the chromosomal fragile site FRA16D tumour suppressor gene, functions in aerobic metabolism and regulates reactive oxygen species. Hum Mol Genet. 2011;20(3):497–509. 10.1093/hmg/ddq495 PMC3016910 21075834

[11] Choo A, O’Keefe LV, Lee CS, et al Tumor suppressor WWOX moderates the mitochondrial respiratory complex. Genes Chromosomes Cancer. 2015;54:745–761. 10.1002/gcc.22286 26390919

[12] Bednarek AK, Keck-Waggoner CL, Daniel RL, et al WWOX, the FRA16D gene, behaves as a suppressor of tumor growth. Cancer Res. 2001;61(22):8068–8073. 11719429

[13] Bednarek AK, Laflin KJ, Daniel RL, Liao Q, Hawkins KA, Aldaz CM. WWOX, a novel WW domain-containing protein mapping to human chromosome 16q23.3-24.1, a region frequently affected in breast cancer. Cancer Res. 2000;60(8):2140–2145. 10786676

[14] Hsu CY, Lee KT, Sun TY, et al WWOX and its binding proteins in neurodegeneration. Cells. 2021;10(7):1–17. 10.3390/cells10071781 PMC8304785 34359949

[15] Lee JW, Bae SH, Jeong JW, Kim SH, Kim KW. Hypoxia-inducible factor (HIF-1)α: Its protein stability and biological functions. Exp Mol Med. 2004;36(1):1–12. 10.1038/emm.2004.1 15031665

[16] Abu-Remaileh M, Aqeilan RI. Tumor suppressor WWOX regulates glucose metabolism via HIF1α modulation. Cell Death Differ. 2014;21(11):1805–1814. 10.1038/cdd.2014.95 PMC4211377 25012504

[17] Mertens J, Herdy JR, Traxler L, et al Age-dependent instability of mature neuronal fate in induced neurons from Alzheimer’s patients. Cell Stem Cell. 2021;28(9):1533–1548.e6. 10.1016/j.stem.2021.04.004 PMC8423435 33910058

[18] Traxler L, Herdy JR, Stefanoni D, et al Warburg-like metabolic transformation underlies neuronal degeneration in sporadic Alzheimer’s disease. Cell Metab. 2022;34(9):1248–1263.e6. 10.1016/j.cmet.2022.07.014 PMC9458870 35987203

[19] Crowther DC, Kinghorn KJ, Miranda E, et al Intraneuronal Aβ, non-amyloid aggregates and neurodegeneration in a *Drosophila* model of Alzheimer’s disease. Neuroscience. 2005;132(1):123–135. 10.1016/j.neuroscience.2004.12.025 15780472

[20] Davie K, Janssens J, Koldere D, et al A single-cell transcriptome atlas of the aging *Drosophila* brain. Cell. 2018;174(4):982–998.e20. 10.1016/j.cell.2018.05.057 PMC6086935 29909982

[21] Georgiev P, Chlamydas S, Akhtar A. *Drosophila* dosage compensation males are from Mars, females are from Venus. Fly (Austin). 2011;5(2):147–154. 10.4161/fly.5.2.14934 PMC3127063 21339706

[22] Donelson N, Kim EZ, Slawson JB, Vecsey CG, Huber R, Griffith LC. High-resolution positional tracking for long-term analysis of *Drosophila* sleep and locomotion using the “tracker” program. PLoS One. 2012;7(5):e37250. 10.1371/journal.pone.0037250 PMC3352887 22615954

[23] Vecsey CG, Koochagian C, Porter MT, Roman G, Sitaraman D. Analysis of sleep and circadian rhythms from Drosophila activity-monitoring data using SCAMP. Cold Spring Harb Protoc 2024;2024:db.prot108182. 10.1101/pdb.prot108182 PMC11552080 38336392

[24] Hendricks JC, Finn SM, Panckeri KA, et al Rest in *Drosophila* is a sleep-like state. Neuron. 2000;25:129–138. 10.1016/s0896-6273(00)80877-6 10707978

[25] Shaw PJ, Cirelli C, Greenspan RJ, Tononi G. Correlates of sleep and waking in *Drosophila melanogaster*. Science. 2000;287(5459):1834–1837. 10.1126/science.287.5459.1834 10710313

[26] Hunt RJ, Granat L, McElroy GS, Ranganathan R, Chandel NS, Bateman JM. Mitochondrial stress causes neuronal dysfunction via an ATF4-dependent increase in L-2-hydroxyglutarate. J Cell Biol. 2019;218(12):4007–4016. 10.1083/jcb.201904148 PMC6891100 31645461

[27] Bolger AM, Lohse M, Usadel B. Trimmomatic: A flexible trimmer for illumina sequence data. Bioinformatics. 2014;30:2114–2120. 10.1093/bioinformatics/btu170 PMC4103590 24695404

[28] Liao Y, Smyth GK, Shi W. FeatureCounts: An efficient general purpose program for assigning sequence reads to genomic features. Bioinformatics. 2014;30(7):923–930. 10.1093/bioinformatics/btt656 24227677

[29] Dobin A, Davis CA, Schlesinger F, et al STAR: Ultrafast universal RNA-seq aligner. Bioinformatics. 2013;29(1):15–21. 10.1093/bioinformatics/bts635 PMC3530905 23104886

[30] Varet H, Brillet-Guéguen L, Coppée JY, Dillies MA. SARTools: A DESeq2- and edgeR-based R pipeline for comprehensive differential analysis of RNA-Seq data. PLoS One. 2016;11(6):e0157022. 10.1371/journal.pone.0157022 PMC4900645 27280887

[31] Stephens M . False discovery rates: A new deal. Biostatistics. 2017;18(2):275–294. 10.1093/biostatistics/kxw041 PMC5379932 27756721

[32] Love MI, Huber W, Anders S. Moderated estimation of fold change and dispersion for RNA-seq data with DESeq2. Genome Biol. 2014;15(12):550. 10.1186/s13059-014-0550-8 PMC4302049 25516281

[33] Wickham H . Ggplot2: Elegant graphics for data analysis. Springer-Verlag New York; 2016.

[34] Raudvere U, Kolberg L, Kuzmin I, et al G:Profiler: A web server for functional enrichment analysis and conversions of gene lists (2019 update). Nucleic Acids Res. 2019;47(W1):W191–W198. 10.1093/nar/gkz369 PMC6602461 31066453

[35] Shen L. GeneOverlap: Test and visualize gene overlaps. R package version 1.49.0. Bioconductor. Icahn School of Medicine at Mount Sinai. 2026.

[36] Gloaguen Y, Morton F, Daly R, et al PiMP my metabolome: An integrated, web-based tool for LC-MS metabolomics data. Bioinformatics. 2017;33(24):4007–4009. 10.1093/bioinformatics/btx499 PMC5860087 28961954

[37] Pang Z, Zhou G, Ewald J, et al Using MetaboAnalyst 5.0 for LC-HRMS spectra processing, multi-omics integration and covariate adjustment of global metabolomics data. Nat Protoc. 2022;17:1735–1761. 10.1038/s41596-022-00710-w 35715522

[38] Creek DJ, Jankevics A, Burgess K, Breitling R, Barrett MP. IDEOM: An excel interface for analysis of LC–MS-based metabolomics data. Bioinformatics. 2012;28(7):1048–1049. 10.1093/bioinformatics/bts069 22308147

[39] Hu Y, Flockhart I, Vinayagam A, et al An integrative approach to ortholog prediction for disease-focused and other functional studies. BMC Bioinformatics. 2011;12:357. 10.1186/1471-2105-12-357 PMC3179972 21880147

[40] Marcelo Aldaz C, Hussain T. Wwox loss of function in neurodevelopmental and neurodegenerative disorders. Int J Mol Sci. 2020;21(23):1–22. 10.3390/ijms21238922 PMC7727818 33255508

[41] O’Keefe LV, Lee CS, Choo A, Richards RI. Tumor suppressor WWOX contributes to the elimination of tumorigenic cells in *Drosophila melanogaster*. PLoS One. 2015;10(8):1–19. 10.1371/journal.pone.0136356 PMC4547717 26302329

[42] Li H, Chawla G, Hurlburt AJ, et al *Drosophila* larvae synthesize the putative oncometabolite L-2-hydroxyglutarate during normal developmental growth. Proc Natl Acad Sci U S A. 2017;114(6):1353–1358. 10.1073/pnas.1614102114 PMC5307464 28115720

[43] Niccoli T, Kerr F, Snoeren I, et al Activating transcription factor 4-dependent lactate dehydrogenase activation as a protective response to amyloid beta toxicity. Brain Commun. 2021;3(2):1–13. 10.1093/braincomms/fcab053 PMC8093921 33977265

[44] Hetz C, Mollereau B. Disturbance of endoplasmic reticulum proteostasis in neurodegenerative diseases. Nat Rev Neurosci. 2014;15(4):233–249. 10.1038/nrn3689 24619348

[45] Souid S, Lepesant JA, Yanicostas C. The xbp-1 gene is essential for development in *Drosophila*. Dev Genes Evol. 2007;217(2):159–167. 10.1007/s00427-006-0124-1 17206451

[46] Lee MH, Shih YH, Lin SR, et al Zfra restores memory deficits in Alzheimer’s disease triple-transgenic mice by blocking aggregation of TRAPPC6AΔ, SH3GLB2, tau, and amyloid β, and inflammatory NF-κB activation. Alzheimers Dement. 2017;3(2):189–204. 10.1016/j.trci.2017.02.001 PMC5651433 29067327

[47] Kim S, Kang SW, Kim SE, et al Genome-wide identification and functional validation of the WW domain containing oxidoreductase gene associated with sleep duration. Sci Rep. 2025;15(1):5552. 10.1038/s41598-024-81158-8 PMC11828923 39952983

[48] Hill VM, O’Connor RM, Sissoko GB, et al A bidirectional relationship between sleep and oxidative stress in *Drosophila*. PLoS Biol. 2018;16(7):e2005206. 10.1371/journal.pbio.2005206 PMC6042693 30001323

[49] Huang SS, Chang NS. Phosphorylation/de-phosphorylation in specific sites of tumor suppressor WWOX and control of distinct biological events. Exp Biol Med. 2018;243(2):137–147. 10.1177/1535370217752350 PMC5788152 29310447

[50] Ju YES, Lucey BP, Holtzman DM. Sleep and Alzheimer disease pathology-a bidirectional relationship. Nat Rev Neurol. 2014;10(2):115–119. 10.1038/nrneurol.2013.269 PMC3979317 24366271

[51] Ju YES, McLeland JS, Toedebusch CD, et al Sleep quality and preclinical Alzheimer disease. JAMA Neurol. 2013;70(5):587–593. 10.1001/jamaneurol.2013.2334 PMC3676720 23479184

[52] Nagarajan P, Winkler TW, Bentley AR, et al A large-scale genome-wide study of gene-sleep duration interactions for blood pressure in 811,405 individuals from diverse populations. Mol Psychiatry. 2025;30(8):3660–3672. 10.1038/s41380-025-02954-w PMC12683674 40181193

[53] Liguori C, Chiaravalloti A, Sancesario G, et al Cerebrospinal fluid lactate levels and brain [18F]FDG PET hypometabolism within the default mode network in Alzheimer’s disease. Eur J Nucl Med Mol Imaging. 2016;43(11):2040–2049. 10.1007/s00259-016-3417-2 27221635

[54] Liguori C, Stefani A, Sancesario G, Sancesario GM, Marciani MG, Pierantozzi M. CSF lactate levels, τ proteins, cognitive decline: A dynamic relationship in Alzheimer’s disease. J Neurol Neurosurg Psychiatry. 2015;86(6):655–659. 10.1136/jnnp-2014-308577 25121572

[55] Long DM, Frame A, Reardon PN, et al Lactate dehydrogenase expression modulates longevity and neurodegeneration in *Drosophila melanogaster*. Aging (Albany NY). 2020;12(11):10041–10058. 10.18632/aging.103373 PMC7346061 32484787

[56] Hussain T, Lee J, Abba MC, Chen J, Marcelo Aldaz C. Delineating WWOX protein interactome by tandem affinity purification-mass spectrometry: Identification of top interactors and key metabolic pathways involved. Front Oncol. 2018;8:1–14. 10.3389/fonc.2018.00591 PMC6300487 30619736

[57] Frame AK, Robinson JW, Mahmoudzadeh NH, Tennessen JM, Simon AF, Cumming RC. Aging and memory are altered by genetically manipulating lactate dehydrogenase in the neurons or glia of flies. Aging (Albany NY). 2023;15(4):947–981. 10.18632/aging.204565 PMC10008500 36849157

[58] Sassano ML, Derua R, Waelkens E, Agostinis P, van Vliet AR. Interactome analysis of the ER stress sensor perk uncovers key components of ER-mitochondria contact sites and Ca^2+^ signalling. Contact. 2021;4:25152564211052392. 10.1177/25152564211052392 PMC10243573 37366380

[59] Vazquez De Aldana CR, Maiton MJ, Hinnebusch AG. GCN20, a novel ATP binding cassette protein, and GCN1 reside in a complex that mediates activation of the elF-2α kinase GCN2 in amino acid-starved cells. EMBO J. 1995;14(13):3184–3199. 10.1002/j.1460-2075.1995.tb07321.x PMC394380 7621831

[60] Devi L, Ohno M. Deletion of the eIF2α kinase GCN2 fails to rescue the memory decline associated with Alzheimer’s disease. PLoS One. 2013;8(10):e77335. 10.1371/journal.pone.0077335 PMC3795630 24146979

[61] Baleriola J, Walker CA, Jean YY, et al Axonally synthesized ATF4 transmits a neurodegenerative signal across brain regions. Cell. 2014;158(5):1159–1172. 10.1016/j.cell.2014.07.001 PMC4149755 25171414

[62] Demay Y, Perochon J, Szuplewski S, Mignotte B, Gaumer S. The PERK pathway independently triggers apoptosis and a Rac1/Slpr/JNK/Dilp8 signaling favoring tissue homeostasis in a chronic ER stress *Drosophila* model. Cell Death Dis. 2014;5(10):e1452. 10.1038/cddis.2014.403 PMC4649510 25299777

[63] Casas-Tinto S, Zhang Y, Sanchez-Garcia J, Gomez-Velazquez M, Rincon-Limas DE, Fernandez-Funez P. The ER stress factor XBP1s prevents amyloid-β neurotoxicity. Hum Mol Genet. 2011;20(11):2144–2160. 10.1093/hmg/ddr100 PMC3090193 21389082

[64] Xiang Y, Xu G, Weigel-Van Aken AK. Lactic acid induces aberrant amyloid precursor protein processing by promoting its interaction with endoplasmic reticulum chaperone proteins. PLoS One. 2010;5(11):e13820. 10.1371/journal.pone.0013820 PMC2972223 21072203

[65] Vlassenko AG, Vaishnavi SN, Couture L, et al Spatial correlation between brain aerobic glycolysis and amyloid-β (Aβ) deposition. Proc Natl Acad Sci U S A. 2010;107(41):17763–17767. 10.1073/pnas.1010461107 PMC2955133 20837517

[66] Bero AW, Yan P, Roh JH, et al Neuronal activity regulates the regional vulnerability to amyloid-β deposition. Nat Neurosci. 2011;14(6):750–756. 10.1038/nn.2801 PMC3102784 21532579

[67] Ježek P . 2-hydroxyglutarate in cancer cells. Antioxid Redox Signal. 2020;33(13):903–926. 10.1089/ars.2019.7902 PMC7533892 31847543

[68] Kalani A, Chaturvedi P, Kalani K, Kamat PK, Chaturvedi P, Tyagi N. A high methionine, low folate and vitamin B6/B12 containing diet can be associated with memory loss by epigenetic silencing of netrin-1. Neural Regen Res. 2019;14(7):1247–1254. 10.4103/1673-5374.251333 PMC6425846 30804256

[69] Pi T, Wei S, Jiang Y, Shi JS. High methionine diet-induced Alzheimer’s disease like symptoms are accompanied by 5-methylcytosine elevated levels in the brain. Behav Neurol. 2021;2021:6683318. 10.1155/2021/6683318 PMC8046555 33880134

[70] Alachkar A, Agrawal S, Baboldashtian M, Nuseir K, Salazar J, Agrawal A. L-methionine enhances neuroinflammation and impairs neurogenesis: Implication for Alzheimer’s disease. J Neuroimmunol. 2022;366:577843. 10.1016/j.jneuroim.2022.577843 PMC13592503 35299077

[71] Baryła I, Kośla K, Bednarek AK. WWOX and metabolic regulation in normal and pathological conditions. J Mol Med. 2022;100(12):1691–1702. 10.1007/s00109-022-02265-5 PMC9691486 36271927

