# Supplementary material for: Alzheimer’s disease risk gene Wwox controls amyloid pathology through metabolic reprogramming
Source: Brain Commun. 2026 Oct 9;8(5):fcag385. doi: 10.1093/braincomms/fcag385 (PMC13684411; doi:10.1093/braincomms/fcag385)
Supplement: Supplementary file 1 [file fcag385_supplementary_data.zip › Supplementary_material.docx]

**Supplementary Material**

**Alzheimer’s disease risk gene *Wwox* controls amyloid pathology through metabolic reprogramming**

**Short title: *Wwox* links metabolism and amyloid**

Hannah J Lucas-Clarke^1,2,†^, Daniel C Maddison^1,2,†^, Leonardo Amadio^1,2,3^, Edgar Buhl^4^, Clement Regnault^5^, Owen Peters^2,3^, James J L Hodge^4^, Gaynor A Smith^1,2^*

1. Division of Psychological Medicine and Clinical Neurosciences, School of Medicine, Cardiff University, CF24 4HQ, UK
2. UK Dementia Research Institute at Cardiff University, Wales, CF24 4HQ, UK
3. School of Biosciences, Cardiff University, Wales, CF10 3AX, UK
4. School of Physiology, Pharmacology and Neuroscience, University of Bristol, BS8 1QU. UK
5. College of Medical Veterinary Life Sciences Shared Research Facilities University of Glasgow, G12 8QQ, UK.

^†^ Hannah J Lucas-Clarke and Daniel C Maddison contributed equally to this work.

*Correspondence to: Gaynor Smith

Hadyn Ellis Building

Cardiff University

Maindy Road

Cardiff

Wales, UK, CF24 4HQ

[Smithga@cf.ac.uk](mailto:Smithga@cf.ac.uk)

# Supplementary Methods

**Quantitative polymerase chain reaction (qPCR)**

Day 0-3 flies were snap-frozen in liquid nitrogen and heads were separated by vortexing (n = 15 heads per N = 3 replicate). RNA was isolated from fly heads homogenised in TRIzol reagent (Invitrogen, 15596026), according to manufacturer’s instructions. Genomic DNA contamination was removed by TURBO DNase kit (Invitrogen, AM1907) and cDNA was synthesised using the QuantiTect Reverse Transcription kit (Thermo Scientific, K0221) according to manufacturer’s instructions. cDNA was amplified using Maxima SYBR Green/ROX master mix (Invitrogen) and the following primers at 300 nM final concentration; *Wwox* forward*: ATGATAGCCCTACCCGACACA or CTTGAGCTCTTTGCGTTCAG, Wwox* reverse: *TGGTTCACATAGCAAACGGTG* or *TGTAGGGCAGTGCGAATACT,* *Ldh* forward: *CGTTTGGTCTGGAGTGAACA*, *Ldh* reverse: *GCAGCTCGTTCCACTTCTCT*, Aβ_42_ forward*: CGATCCTTCTCCTGCTAACC,* Aβ_42_ reverse*:* *CACCATCAAGCCAATAATCG, rp49* forward: *AGCATACAGGCCCAAGATCG, rp49* reverse: *TGTTGTCGATACCCTTGGGC.* The QuantStudio 7 thermocycler was used with the following conditions: Initial denaturation (95 ^o^C, 600 seconds), denaturation (95 ^o^C, 15 seconds), annealing (60 ^o^C, 30 seconds), extension (72 ^o^C, 30 seconds) (40 cycles), melt curve (60-95 ^o^C). Melt curve analysis was performed to ensure single-product amplification. Raw amplification data was processed using the qpcR package in RStudio.^1^ The fold-change ratio of *rp49*-normalised target gene expression between each experimental line and its genetic background control was calculated using the *ratiobatch* function. Statistical significance was calculated using a pairwise fixed random reallocation test: efficiency values tied to Cp values are randomly shuffled between experimental and control samples for 1000 permutations. A fold-change ratio is calculated for each permutation and compared to non-shuffled value. The number of permutations which produce a fold-change greater than, equal to or smaller than the original data is used to generate the p value.

**Alternatively spliced Xbp1 imaging**

We utilised the fluorescent genetically encoded reporter line *UAS-Xbp1:EGFP:HG* driven pan-neuronally to measure alternatively spliced *Xbp1*. Fresh, unfixed, brains (N = 6-9) from day 14 females were dissected and mounted on bridge slides for immediate imaging on a Zeiss Cell Observer spinning disk confocal with an Axiocam 503 camera, using an excitation wavelength of 488 nm and 20X objective. Four regions of interest were drawn over the brain and mean gray values were calculated, avoiding trachea (FIJI, version 2.3.0). Data was normalised to the average of control and analysed via t-test (GraphPad Prism v. 10) – exploring the effect of *Wwox* knockdown in the presence of amyloid.

**Measurement of ATP concentration**

ATP concentrations were measured via the Celltiter-Glo 2.0 luciferase assay (Promega, G9242) from 14-day old female flies. n = 3 brains were dissected per replicate (N = 4) per genotype and homogenised in 100 μl 0.1 % PTX. Debris was removed via centrifugation and the supernatant was subsequently equilibrated to room temperature (30 minutes) before addition of an equal volume of Celltiter-glo and a further 2 minutes incubation (with rotation). After a further 10 minutes luminescence was measured on a FLUostar Omega plate reader with 520 nm filter and 1.24 second exposure per well. Luminescence was averaged across four replicate wells per genotype and background luminescence subtracted. Results were then analysed via two-way ANOVA (GraphPad Prism v. 10).

**H_2_O_2_ measurements through roGFP2-Orp1 imaging**

Mitochondrial hydrogen peroxide levels were measured using the ratiometric *UAS-roGFP2-Orp1* reporter. *Drosophila* wings from day 7 females (N = 9-11) were dissected and mounted onto microscope slides. The intersection of Longitudinal vein 1 (LV1) to LV2 was imaged at 63X using a Zeiss spinning disc confocal with Z-stacks with width 1 μm. Oxidised roGFP2-Orp1 was excited at 405 nm and the reduced form at 488 nm. The images were thresholded based on the reduced channel and intensity measured within an ROI in the vein and reported as 405/488 normalised to control. The ratios were then analysed using two-tailed unpaired t-test.

# Supplementary Figures

**Supplementary Figure 1**

**Supplementary Figure 1. qPCR of *Wwox* mRNA levels from fly heads upon *elav-GAL4* driven expression of *Wwox* targeting TRiP RNAi for knockdown, or gRNA for CRISPRa upregulation.** *Wwox* mRNA levels were normalised to *rp49* housekeeping gene and each group was also normalised to its corresponding control genotype. Mean ± SD. Statistics = pairwise fixed reallocation randomization test, *** p < 0.001, N = 3 replicates (15 heads per n), newly eclosed females. Datapoints represent each biological replicate.

Alt text: Bar graph of qPCR results, genotype (x) versus *Wwox* mRNA levels (y), reveals significant down regulation with RNAi and upregulation with gRNA.

**Supplementary Figure 2**

**Supplementary Figure 2. *Wwox* manipulation in neurons, glia or the clock decreases activity and increases sleep.** Neuronal knockdown of *Wwox* decreases **A)** total activity counts in the light (t = 8.665, p_adj_ < 0.0001) and dark (t = 5.416, p_adj_ < 0.0001), **B)** maximum wake episode duration (F = 49.46, p_adj_ < 0.0001) and **C)** mean wake episode duration (light t = 6.852 p_adj_ < 0.0001, dark t = 2.866 p_adj_ = 0.0128). **D)** The number of sleep episodes decreases in the dark (t = 4.782, p_adj_ < 0.0001) after neuronal knockdown of *Wwox* **E)** whereas the time taken to fall asleep is shorter in the light (t = 5.73, p_adj_ < 0.0001) and dark (t = 2.358, p_adj_ = 0.0490). Similar effects of activity are seen after glial knockdown of *Wwox*. **F)** Total activity decreases (light t = 6.263 p_adj_ < 0.0001 and dark t = 2.389 p_adj_ = 0.0444), **G)** max wake episode is shortened (F = 53.07, p_adj_ < 0.0001), **H)** and mean wake episode is shorter after *Wwox* knockdown during the light phase only (t = 5.677, p_adj_ < 0.0001). **I)** The number of sleep episodes is increased in the light phase (t = 2.980, p_adj_ = 0.0107) and **J)** the latency to fall asleep is shorter in both the light (t = 6.771, p_adj_ < 0.0001) and dark (t = 3.687, p = 0.0011). Clock knockdown of *Wwox* decreases **K)** activity counts normalized to time awake (F = 64.82, p_adj_ < 0.0001), **L)** increases probability to sleep in the dark (t = 4.186, p_adj_ = 0.0002), and **M)** increases mean sleep episode duration in the dark (t = 3.84, p_adj_ = 0.0006). **N)** Clock knockdown decreases total activity in the dark (t = 6.079, p_adj_ < 0.0001), **O)** and max wake episode duration (F = 5.203, p_adj_ = 0.0403). However, no effects were seen on **P)** mean wake episode duration (F = 0.5447, p_adj_ = 0.5272), **Q)** number of sleep episodes (F = 2.420, p_adj_ = 0.1940) or **R)** latency to sleep (F = 0.8357, p_adj_ = 0.4339). **S)** Circadian rhythmicity remained strong after clock knockdown of *Wwox* (t = 3.205, p = 0.0018) and **T)** circadian period was significantly lengthened (U = 820.5, p = 0.0006). All sleep and circadian experiments were performed on male flies 0-6 days entering the DAM apparatus. Circadian rhythmicity was analysed on a smaller N due to removal of deaths, and circadian period was assessed after further removal of arrhythmic flies. **U**) This increase in sleep may be due to increased mitochondrial hydrogen peroxide levels as assessed using roGFP2-Orp1 (405-oxidised Orp1, 488-reduced) after neuronal knockdown of *Wwox* in day 7 female wings. Statistics = Two-way ANOVAs for genotype and light phase were performed followed by Bonferroni multiple comparison tests if the interaction term was significant (Panels A-R). P values from the ANOVA and Bonferroni tests were then corrected by 5 % FDR to account for the number of metrics being assessed (full results summarised in Supplementary Table 2). Unpaired t-tests were performed in S and U and Mann-Whitney test in T. Dotted lines in panels S represent RS = 1 (weakly rhythmic threshold), RS = 1.5 (strongly rhythmic threshold) and panel T period length = 24 hours. p/p_adj_ * < 0.05, ** < 0.01, *** < 0.001, **** < 0.0001. Data points represent individual flies: A-E N = 31-80, F-J N = 53-56, K-R N = 53-61, S N = 48-60, T N = 47-57, U N = 9-11 per genotype. Mean ± SD. Scale bar = 20 μm.

Alt text: Graphs summarising sleep and circadian results and ROS production after *Wwox* knockdown, labelled A to U. A to R are bar graphs of sleep metrics. S and T are bar graphs of circadian metrics. U shows 4 representative images of the wing with ROS reporter, and a bar graph quantifying to the right.

**Supplementary Figure 3**

**Supplementary Figure 3. Knockdown of *Wwox* in neurons or glia whilst simultaneously expressing Aβ_42_ in neurons is detrimental to CNS health. A)** qPCR of Aβ_42_ transcript levels reveals no differences in transcripts relative to *rp49* in newly eclosed *Wwox* knockdown (p = 0.2565) and overexpressing (p = 0.3110) heads normalised to their corresponding genetic background controls. (N = 3 replicates, 15 heads per n, pairwise fixed random reallocation test, mean ± SD). **B)** Representative images of startle induced locomotion over a ten second (s) period after neuronal *Wwox* knockdown in the presence of Aβ_42_ compared to control (21 days old). **C)** *Wwox* knockdown in glia, whilst expressing amyloid in neurons (*nsyb-QF2 > QUAS-Aβ_42_),* decreased lifespan (χ^2^ = 6.720 p = 0.0095, Mantel-Cox tests N = 100-115 individual flies ** p < 0.01.), **D)** but had no effect on locomotion (t = 0.9876 p = 0.3491, unpaired t-test, N = 5-6 vials of n = 5-10 flies, mean ± SD) at day 7. **E)** Immunostaining of Aβ_42_ in *elav-Gal4 > LacZ* brains at day 14 is negative for signal. Scale bar = 100 µm. All experiments performed on females. Datapoints represent N: individual biological replicates (panel A), and independent vials (panel D). ns = non-significant.

Alt text: Graphs and representative images labelled A to E. A is a bar graph of qPCR results. B is representative images of flies climbing in vials in control but not in knockdown condition at day 21 across ten second timepoints. C is a Kaplan-Meier plot (age versus percent survival). D is a bar graph. E is a representative image.

**Supplementary Figure 4**

**Supplementary Figure 4. Principal component analysis shows clear separation of genotypes.** Genotypes from neuronal knockdown of *Wwox* transcriptomics experiment separate along **A)** PC1 (23 %) vs. PC2 (15 % variance). N = 4 replicates from n = 10 pooled heads (day 14 females). Abbreviations: PC = Principal component.

Alt text: PCA plot coloured by genotype (four points per genotype) with PC1 on x axis and PC2 on y axis.

**Supplementary Figure 5**

**Supplementary Figure 5. Aβ_42_-dependent increase in Atf4 upon *Wwox* knockdown is not accompanied by changes in ATP levels or Xbp1-splicing. A)** Log_2_ fold-changes were plotted for the effect of *Wwox* knockdown in a LacZ background or Aβ_42_ background_,_ revealing differing directions of L-Lactate concentrations with *Wwox* knockdown (N = 5 replicates of n = 10 pooled heads, day 14 females). Datapoints represent individual metabolites. **B)** These changes in Lactate however are not accompanied by any differences in ATP concentrations (Celltitre-glo) after *Wwox* knockdown (statistics = two-way ANOVA F = 0.3376 p = 0.5720), data is presented normalised to control, mean ± SD, N = 4 replicates (n = 3 brains per sample, averaged across four technical replicate wells, day 14 females). Datapoints represent biological replicates. **C)** qPCR of *Ldh* mRNA levels from newly eclosed fly heads upon *elav-GAL4* driven expression of *Ldh* targeting RNAi combined with Aβ_42_ expression. *Ldh* mRNA levels were normalised to *rp49* housekeeping gene and each group was also normalised to its corresponding control genotype. Mean ± SD. Statistics = pairwise fixed reallocation randomization test, * p < 0.05, N = 3 replicates (15 female heads per n, day 14 females). Datapoints represent replicates. **D)** Atf4 immunocytochemistry in day 14 female fly brains **E)** demonstrated no upregulation of Atf4 in the brain after *Wwox* knockdown in the absence of Aβ_42_ (mean ± SD, N = 6 brains, statistics = two-tailed unpaired t-test t = 0.0894 p = 0.9305). Datapoints represent independent brains. **F-G)** There were no differences in the levels of *Xbp1:GFP:HG* (reporter which only fluoresces when *Xbp1* is alternatively spliced) after *Wwox* knockdown in the presence of Aβ_42_ (t = 1.365 p = 0.1954, data is presented normalised to control, day 14 females). Mean ± SD. Statistics = two-tailed unpaired t-test, N = 6-9 brains, datapoints represent independent brains. Representative images, scale bar = 100 μm.

Alt text: Graphs and representative images labelled A to G. A is a scatter plot with dotted lines at the origin splitting the graph into quadrants. L-lactate is the only significant metabolite labelled in the top left quadrant. B, C, E and G are bar plots with genotypes on the x axis. D and F are representative images.

**Supplementary Figure 6**

**Supplementary Figure 6. Overexpression of *Wwox* in control neurons or overexpression of *Wwox* in glia whilst expressing neuronal Aβ_42_ has no effect on behavior. A)** There is no difference between the survival curves of flies expressing control^gRNA^ or *Wwox*^gRNA^ for CRISPRa overexpression in neurons (*elav-GAL4*) in a control background (statistics = Mantel-Cox test χ^2^ = 2.156 p = 0.1420, N = 76-86 individual female flies). **B)** Distance climbed after 4 seconds (s) in a startle induced locomotion assay Startle-induced locomotion of flies expressing neuronal control^gRNA^ or *Wwox*^gRNA^ revealed no effect of genotype (statistics = two-way ANOVA F = 0.024 p = 0.8770, N = 3-6 replicate vials of n = 5-10 flies, days 7-28 females). **C)** Expressing *hWWOX* into neurons also expressing Aβ_42_ could significantly rescue lifespan (statistics = Mantel-Cox test χ^2^ = 9.493 p = 0.0021, N = 85-106 individual female flies). **D)** Climbing (days 7-28 females) was significantly improved by expressing *hWWOX* (q = 7.256, p_adj_ < 0.0001), *dWwox* (q = 12.29, p_adj_ < 0.0001), *WWOX* pSer14 mimic (q = 10.2, p_adj_ < 0.0001), *WWOX* pTyr33 mutant (q = 9.071, p_adj_ < 0.0001) or *WWOX* pSer14 mutant (q = 4.411, p_adj_ < 0.0281) into Aβ_42_ expressing flies. Expression of *dWwox* outperformed *hWWOX* (q = 5.032, p_adj_ = 0.0075) and *WWOX* pSer14 mutant flies (q = 7.878, p_adj_ < 0.0001). *WWOX* pSer14 mimic climbed significantly better than *WWOX* pSer14 mutant flies (q = 5.794, p_adj_ = 0.0012). *WWOX* pTyr33 mutant flies outperformed *WWOX* pSer14 mutant (q = 4.661, p_adj_ = 0.0168). Statistics = two-way ANOVA followed by Tukey’s multiple comparisons (N = 5 vials of n = 5-10 flies). **E)** However, overexpression of *Wwox* (*Drosophila* or human) in glial cells, whilst expressing Aβ_42_ in neurons, had no effect on lifespan (statistics = Mantel-Cox test correcting for multiple comparisons with 5 % FDR χ^2^ = 0.0649 p_adj_ = 0.8000, χ^2^ = 3.768 p_adj_ = 0.1045, N = 84-154 individual female flies) or **F)** startle induced locomotion (statistics = one-way ANOVA F = 0.4194 p = 0.6654, N = 4-7 replicate vials of n = 5-10 flies, day 7 females) compared to an empty vector control. **G)** The MSD assay for amyloid revealed decreased total amyloid after *Wwox* overexpression (statistics = unpaired two-tailed t-test t = 2.352, p = 0.0313), **H)** but no difference in the ratio of insoluble to soluble species (statistics = unpaired two-tailed t-test t = 1.334 p = 0.2530, N = 3 replicates of n = 25 heads, day 14 females). p/p_adj_ * < 0.05, ** < 0.01, *** < 0.001, **** < 0.0001 ns = non-significant. Datapoints represent N: independent vials (panels B, D and F) and independent replicates of pooled heads (panels G-H).

Alt text: Graphs labelled A to H describing *Wwox* overexpression results. A, C and E are Kaplan-Meier plots (age versus percent survival). B and D are line graphs of climbing (age versus distance climbed after four seconds in centimetres). F to H are bar graphs (genotype on x axis).

**Supplementary Figure 7**

**Supplementary Figure 7. Overexpression of *Wwox* in the presence of Aβ_42_** **differentially expresses genes associated with synapses, metabolism and immune responses.** **A)** Principal component analysis reveals that 29 % of the variation in the dataset can be explained by PC1 and 16 % by PC2 (datapoints represent N = 3-4 independent replicates of n = 10 pooled day 14 female heads). **B)** Overrepresentation analysis (g:Profiler) of the effect of *Wwox* overexpression in a LacZ background revealed enrichment of fatty acid metabolism. **C)** Overrepresentation analysis of the effect of Aβ_42_ revealed enrichment for metabolic terms. **D)** The genes differentially expressed by Aβ_42_ in the *Wwox* knockdown and overexpression experiments were significantly similar (statistics = Fisher Exact Test GeneOverlap package). Heatmap of log_2_ fold-change the nine shared genes in both experiments reveals that eight genes change in the same direction. **E)** Overrepresentation analysis for the effect of *Wwox* overexpression in an Aβ_42_ background reveals immune, synaptic and metabolic pathway enrichment. Datapoints in overrepresentation analysis represent individual pathways. Abbreviations: PC = Principal component, KD = Knockdown, OE = Overexpression, OR = Odds Ratio, LFC = Log_2_ fold-change GO:BP = Gene ontology biological processes, GO:MF = Gene ontology molecular functions, KEGG = Kyoto Encyclopedia of Genes and Genomes, TF = TRANSFAC, WP = Wiki pathways, LFC = log_2_ fold-change.

Alt text: Graphs summarising transcriptional changes after *Wwox* overexpression, labelled A to E. A is a PCA plot (PC1 versus PC2) coloured by genotype. B, C and E are dot plots summarising pathway analysis results, separated by database. D is a venn diagram of genes with heatmap (blue to red) of log_2_ fold-change below, from knockdown and overexpression experiments.

**Supplementary Figure 8**

**Supplementary Figure 8. L-methionine levels differ with *Wwox* overexpression dependent on Aβ_42_ status.** Relative abundance of two (panels A-B) individually standard matched L-methionine metabolites after *Wwox* overexpression in the presence or absence of Aβ_42_ (two-way ANOVA followed by multiple testing correction with 10 % FDR). Interaction term: (panel A) F = 19.79 p_adj_ = 0.0073 and (panel B) F = 14.92 p_adj_ = 0.0133). Data points represent N = 5 replicates of n = 10, day 14 female pooled heads. Mean ± SEM.

Alt text: Two bar graphs labelled A and B summarising the levels of L-methionine across the four genotypes (LacZ, AB42 with or without overexpression of *Wwox*).

**Supplementary Tables**

Uploaded in a separate file with this manuscript.

**Supplementary Table 1A.** Stock genotypes and sources

**Supplementary Table 1B.** Progeny genotype, sex, and age per assay

**Supplementary Table 2.** Complete correlation matrix and ANOVA results for sleep experiments associated with Figure 2 and Supplementary Figure 2.

**Supplementary Table 3.** Differentially expressed genes and overrepresentation analysis (*Wwox* knockdown experiment)

**Supplementary Table 4.** Differentially expressed metabolites (*Wwox* knockdown experiment)

**Supplementary Table 5**. Differentially expressed genes and overrepresentation analysis (*Wwox* overexpression experiment)

**Supplementary Table 6.** Log_2_ fold-changes and overrepresentation analysis of genes commonly changed by *Wwox* overexpression and amyloid

**Supplementary Table 7.** Differentially expressed metabolites (*Wwox* overexpression experiment)

**Supplementary References**

1.	Ritz C, Spiess AN. qpcR: An R package for sigmoidal model selection in quantitative real-time polymerase chain reaction analysis. *Bioinformatics*. 2008;24(13):1549-1551. doi:10.1093/bioinformatics/btn227
